# High-resolution thermal imaging to delineate effects of cancer-induced aerobic glycolysis and endothelial dysfunction

**DOI:** 10.1093/noajnl/vdag007

**Published:** 2026-01-18

**Authors:** Daniel Coman, Peter Herman, Jyotsna U Rao, Jelena Mihailovic, Yuegao Huang, Gen Kaneko, Fahmeed Hyder

**Affiliations:** Department of Radiology & Biomedical Imaging, Yale University, New Haven; Department of Biomedical Engineering, Yale University, New Haven; Department of Radiology & Biomedical Imaging, Yale University, New Haven; Department of Radiology & Biomedical Imaging, Yale University, New Haven; Department of Radiology & Biomedical Imaging, Yale University, New Haven; Department of Radiology & Biomedical Imaging, Yale University, New Haven; Department of Radiology & Biomedical Imaging, Yale University, New Haven; Department of Radiology & Biomedical Imaging, Yale University, New Haven; Department of Biomedical Engineering, Yale University, New Haven

**Keywords:** acidity, aerobic glycolysis, blood flow, endothelial dysfunction, temperature

## Abstract

**Background:**

Glioblastoma multiforme (GBM) is an aggressive brain tumor with abysmal prognosis because cancer cell growth in the tumor microenvironment (TME) is orchestrated by complex interplay between aerobic glycolysis (AG) and endothelial dysfunction (ED). AG acidifies extracellular pH (pH_e_) to promote tumor invasion and suppress immune response, whereas ED leads to leaky blood vessels which hampers perfusion and stimulates hypoxia. Since metabolism generates heat and perfusion removes heat, we hypothesized that temperature could reflect both metabolic and vascular reprogramming in the TME mediated by AG and ED.

**Methods:**

We used multiple magnetic resonance methods and bioheat modeling to dissect temperature contributions from metabolic and vascular sources in rat gliomas.

**Results:**

Upregulated AG in the TME results from enhanced glycolysis (∼4.2× higher) and reduced glucose oxidation (∼4.8× lower), which leads to more acidic pH_e_ (6.9 ± 0.1 vs 7.3 ± 0.1). Since TME is hypoperfused (∼40% lower) and glycolysis is less exothermic compared to glucose oxidation, simulations predict a cooler TME as *in vivo* measurements clearly demonstrate (0.5-1.5 °C). Moreover, temperature and pH_e_ are correlated both inside and outside the TME for untreated and treated rats (*r* > 0.6).

**Conclusions:**

Since TME is more glycolytic, acidic, hypoperfused, and cooler than neighboring milieu, thermal mapping can represent combined effects of AG and ED for early GBM detection and therapy optimization.

Key Points

- Glioma is not only more glycolytic and acidic, but also hypoperfused and cooler than normal tissue.
- Thermal mapping represents the combined effects of aerobic glycolysis and ­endothelial dysfunction, which are major cancer hallmarks.

Importance of the Study

As confirmed in this study, and as previous studies suggest, there is uncoupling between blood flow and glucose oxidation in the tumor microenvironment which could affect local temperature. Our hypothesis is that because of uncoupling between blood flow and oxidative metabolism, thermoregulation might be deficient in the tumor and which results in temperature differences between tumor and normal tissue. There is paucity of studies that investigate the temperature of tumors in relation to normal brain. This study shows that the lower temperature observed in tumors reflects combined contributions from aerobic glycolysis and endothelial dysfunction. While temperature is rarely included in discussions related to studies of tumor microenvironment, our results implicate future therapeutic directions because cooler conditions have been shown to enhance tumor growth. Since evidence suggests that temperature regulates the immune response, thermal-therapy and immuno-therapy may be combined for improved outcome. Thus, thermal mapping has translational potential involving early tumor detection, treatment planning and monitoring or optimization of hyperthermic ablation.

Glioblastoma multiforme (GBM) is a very aggressive brain tumor with poor prognosis, high resistance to chemotherapy, and short survival rates.^1^ Tumors have physicochemical properties significantly different from normal tissue.^2^ Cancer cells reshape metabolism to favor biosynthetic pathways (glycolytic) over energetic pathways (oxidative) such that tumor growth (ie, proliferation, invasion) continues in harsh conditions while building resistance to treatments.^3^ Thus, metabolic reprogramming allows the tumor microenvironment (TME) to thrive while providing energy through glycolysis-derived adenosine triphosphate (ATP) even in the presence of sufficient oxygen (ie, aerobic glycolysis or Warburg effect),^4^ a process which results in overproduction of acids that need to be extruded thereby acidifying the extracellular space to promote tumor invasion and suppress immune response.^5^ Under normal conditions endothelial cells act as stern gatekeepers of brain health, but in GBMs the vasculature is reprogrammed also to form immature blood vessels which are leaky and thus perfusion is hindered to induce hypoxia.^6^ Since blood flow removes heat and metabolism generates heat,^7^ these components regulate tissue temperature. To dissect complexities underlying the interplay between aerobic glycolysis (AG) and endothelial dysfunction (ED) in the TME, there is need for measurements of cerebral blood flow (CBF), cerebral metabolic rates of glucose (CMR_glc_) and oxygen (CMR_O2_) consumption because they collectively regulate extracellular pH (pH_e_) and temperature.

In the normal brain there is tight coupling between CBF, CMR_glc_, and CMR_O2_.^8^ The degree of AG is inferred from the oxygen to glucose index (OGI) which is defined as,


(1a) $$\text{OGI}={\text{CMR}}_{\text{O}2}/{\text{CMR}}_{\text{glc}}$$

An OGI maximum of 6 means glucose is fully oxidized generating carbon dioxide and water with abundant oxidative ATP generation, whereas an OGI of less than 6 indicates some level of AG with overproduction of acids and underproduction of oxidative ATP.^9^ Similarly, the ED can be reflected by the oxygen extraction fraction (OEF),


(1b) $$\text{OEF}={\text{CMR}}_{O2}/\left(C_{a}\;\cdot \;\text{CBF}\right)$$

where C_a_ is the arterial oxygen concentration and OEF links oxygen demand (CMR_O2_) to oxygen supply $\left(C_{a}\;\cdot \;\text{CBF}\right)$ signifying limits of hypoxia and/or ischemia. Since CMR_O2_ and CBF respectively are critical factors of heat generated vs heat removed,^10^ changes in metabolism,^11^ and perfusion^12^ impact the temperature based on bioheat transfer theory.^13^ In cancer, however, AG implies uncoupling between CMR_glc_ and CMR_O2_ to lower OGI,^14^ and ED suggests a mismatch between CBF and CMR_O2_ to alter OEF.^15^ While it is well known that pH_e_ in the TME is acidic (eg, murine brain tumors,^16^^,^^17^ rabbit liver tumors^18^^,^^19^), we hypothesize that temperature in the TME is altered because metabolism generates heat and perfusion removes heat, as hinted by previous studies.^20^^,^^21^ Since cancer cell growth in GBM is regulated by metabolic and vascular reprogramming, we also posit that temperature and pH_e_ in the TME are correlated. To test these hypotheses, we measured CBF, CMR_glc_, CMR_O2_, temperature, and pH_e_ in rat brains with 4 glioma lines (9L, RG2, U87, and U251) using magnetic resonance imaging (MRI) and spectroscopic imaging (MRSI) methods.

## Methods

### Tumor Models, Tumor Implantation, and Tumor Treatment

We used 9L, RG2, U87, and U251 tumor lines in adult Fischer 344 rats (220-280 g; *n* = 48) and athymic nude rats (200-250 g; *n* = 16). Rats were kept in temperature- and humidity-controlled rooms with food and water available *ad libitum* for at least 2 weeks prior to scans. Tumor implantation was at 10-12 weeks for Fisher rats and 8-10 weeks for athymic nude rats. MRI and MRSI scanning was at 12-15 weeks for Fisher rats and 10-13 weeks for athymic nude rats. See Methods S1 for details on tumor models, tumor implantation, and tumor treatment.

### Animal Preparation for In Vivo Magnetic Resonance Scans

All animal protocols were approved by the Institutional Animal Care and Use Committee at Yale University. All *in vivo* scans with MRI and MRSI were performed on an 11.7T Bruker horizontal bore spectrometer (Billerica, Massachusetts, United States) with specifically designed radio frequency (RF) coils (Methods S2.1-S2.6).

Transverse relaxation time (*T*_2_) weighted MRI provided anatomical imaging. We measured pH_e_ and temperature with a ^1^H-MRSI technique that combines high spatial resolution of MRI with high molecular specificity of MRS called Biosensor Imaging of Redundant Deviation in Shifts (BIRDS; Methods S2.1). In BIRDS, the paramagnetically-shifted nonexchangeable protons (ie, -CH_y_, *y* = 1,2,3) on DOTA-based macrocyclic complexes are directly detected.^22–24^ Proton chemical shifts emanating from complexes of thulium (Tm^3+^) ion with either 1,4,7,10-tetraazacyclododecane-1,4,7,10-tetrakis(methylenephosphonate) (DOTP^8-^) or 1,4,7,10-tetraazacyclododecane-1,4,7,10-tetramethyl-1,4,7,10-tetraacetate (DOTMA^4-^) are very sensitive to changes in their environment allowing pH_e_ and/or temperature detection with high accuracy.^23^^,^^24^ Proton signals from TmDOTP^5-^ or TmDOTMA^-^ have extremely short relaxation times (<5 ms) allowing high-speed MRSI.^23^^,^^24^ For BIRDS, the pH_e_ was measured with TmDOTP^5-^, whereas temperature was measured with TmDOTP^5-^ and TmDOTMA^-^. For temperature validation, we also used the ^1^H-MRS method based on the chemical shift difference between water and N-acetyl aspartate (NAA).^25^ CMR_O2_ was measured by ^1^H-[^13^C] MRS or proton-observed carbon-edited (POCE) which required infusion of [1,6-^13^C]-D-glucose^26^ to estimate the tricarboxylic acid (TCA) flux (V_TCA_) which is stoichiometrically linked to CMR_O2_ (Methods S2.2). CMR_glc_ was measured by ^19^F-MRSI which required infusion of 2-fluoro-2-deoxy-D-glucose (FDG) where both the glucose analog (FDG) and its phosphorylated product 2-fluoro-2-deoxy-D-glucose-6-phosphate (FDG-6P) could be measured (Methods S2.3).^27^ ED was determined from CBF measured by ^1^H-MRI with pulsed arterial spin labeling (PASL) using longitudinal relaxation time (*T*_1_) with different inversion recovery times (TIRs) (Methods S2.4)^28^ and cerebrovascular reactivity (CVR) experiments (Methods S2.5).^29^ The *T*_1_ maps were not used for TIR selection for CBF because *T*_1_ measurement errors across normal or tumor regions would propagate into additional errors for CBF estimations. Since different *T*_1_ values in tumor and normal tissue is well-established,^28^ and averaging over a larger number of pixels significantly increases the accuracy of *T*_1_ measurement for both tumor and normal tissue, the evident choice is the selection of one TIR for normal tissue and another TIR for tumor tissue. For TME cellularity (or density) estimates we used ^1^H-MRI with apparent diffusion coefficient (ADC) imaging (Methods S2.6).^30^

The rats were anesthetized with isoflurane (<2%), tracheotomized, and artificially ventilated (70% N_2_O/30% O_2_). A femoral vein was cannulated (PE-10) to administer either [1,6-^13^C]-D-glucose (9.4 mmol/kg), FDG (2.7 mmol/kg), TmDOTP^5-^ (0.5 mmol/kg), or TmDOTMA^-^ (0.5 mmol/kg). A femoral artery was cannulated (PE-50) for monitoring physiological parameters (pCO_2_, pO_2_, pH, blood pressure) throughout the experiment. An intraperitoneal line was inserted for administration of α-chloralose (40 mg/kg/hr), which was chosen to be consistent with past BIRDS measurements in rat brain tumors.^23^^,^^24^ For BIRDS, renal ligation was employed to maintain the TmDOTP^5-^ (or TmDOTMA^-^) concentration at ∼5-6 mM in the blood and ∼3-4 mM in the brain’s extracellular space.^23^^,^^24^ Majority of *in vivo* TmDOTP^5-^/TmDOTMA^-^ signals arise from extracellular space because blood represents only ∼3% of voxel volume.^23^^,^^24^ Because body temperature decreases in animals under anesthesia, a heating pad with continuous ­circulating warm water (38-42 °C) was used to maintain body temperature (measured with a rectal probe) in the physiological range (36.5-37.5 °C) for the duration of the experiment.

### Temperature Simulations Using the Steady-State Bioheat Equation

The spatial distribution of temperature was calculated using steady-state Pennes bioheat equation^13^ applied to a 1mm slice positioned near the surface of the brain underneath the skull. In living tissue, the bioheat is composed of metabolic heat production (*Q_m_*), heat contribution due to blood flow (*Q_f_*), heat conductance through tissue (*Q_k_*) and heat loss to the extracranial space environment (*Q_c_*). At steady-state the total heat *Q_k_*+*Q_m_*+*Q_f_*+*Q_c_* is zero (heat production equals heat loss). For a voxel with in-plane coordinates (*x*, *y*), these 4 types of heat are defined as


(2a) $$Q_{m}={\mathrm{CMR}}_{O2}({\Delta H}_{0}-{\Delta H}_{b})+\max\left[({\mathrm{CMR}}_{\mathrm{glc}}-\frac{1}{6}{\mathrm{CMR}}_{O2}),0\right]{\Delta H}_{\mathrm{glyc}}$$

(2b) $$Q_{f}=\rho_{bl}c_{bl}C\mathrm{BF}(T_{\mathrm{art}}-T_{ts}(x,y))$$

(2c) $$Q_{k}=k\frac{1}{\rho_{ts}}\nabla^{2}T_{ts}\left(x,y\right)$$

(2d) $$Q_{c}=hA(T_{\mathrm{env}}-T_{ts}(x,y))$$

where definitions, values, and units are given in Table S1. In Equation (2a), the glycolytic energy production is included in the enthalpy of oxidative phosphorylation. If (stoichiometrically) more glucose than oxygen is consumed, then an additional glycolytic heat (through glycolytic enthalpy ${\Delta H}_{\mathrm{glyc}}$) contributes to metabolic heat production. In Equation (2b) there is an inflow of heat if the arterial blood temperature *T_art_* is higher than the tissue temperature *T_ts_*(*x*, *y*). This model assumes that *T_art_* is the same at every tissue location. The heat exchange with the neighboring voxels is modeled using a conductive heat exchange term (Equation (2c)). In Equation (2d) the heat loss to the extracranial environment does not consider the skin heat regulation. The *Q_c_* term applies to all the voxels (independent of location) since all voxels are positioned close to the skull. The voxel size is 1 mm × 1 mm × 1 mm and the surface area A describing the heat exchange with the extracranial environment in Equation (2d) is *A* = 1 mm × 1 mm which is equal to 10^−6^ m^2^ (Table S1) corresponding to heat transfer only through one side of the voxel, facing the extracranial environment.

The spatial distribution of temperature was calculated by solving the partial differential equations in Matlab (Mathworks Inc., Matick, MA, United States) with the Partial Differential Equation Toolbox. The model simulates the horizontal cross section of the rat brain with a 5 mm dia­meter circular tumor on the right hemisphere. The model assumes a perpendicular heat loss towards/through the skull defined by Equation (2d). A Neumann type boundary condition was used, where the flux at the boundary describes the heat that flows into or out of the system. This provides a more accurate description of heat exchange at the edge of the brain compared to a Dirichlet boundary condition which sets a fixed temperature value at the boundary. *T_art_*, *T_env_*, CMR_O2_, CMR_glc_, and CBF values used in this model were measured experimentally, while other constants were obtained from literature.^7^^,^^31^^,^^32^ Although the heat transfer coefficient between brain tissue and air *h_n_* = 8 J/s/m^2^/g/°C was previously measured,^32^ a corresponding value for brain tumors is not available in the literature. However, based on ∼2.5-fold difference in cellular density in gliomas compared to cortical tissue and because *Q_c_* scales with tissue density, we estimate the heat transfer coefficient of tumor *h_t_* as 2.5h_n_ (Tables S1 and S2). ADC measures show significantly lower values inside tumors compared to normal tissue (Figure S4) indicating a more restricted water diffusion in tumors, consistent with higher cellular density in gliomas.^33^ For temperature simulations we used the ratios between the parameters inside tumor and normal tissue (Table S2).

### Statistics

All results were expressed as mean ± standard deviation and comparisons across groups were assessed by Student’s *t*-test with 2 tails, where *p*-values less than 0.05 were ­considered statistically significant. The error bars shown in figures represent the corresponding standard deviations.

## Results

**Figure 1.**
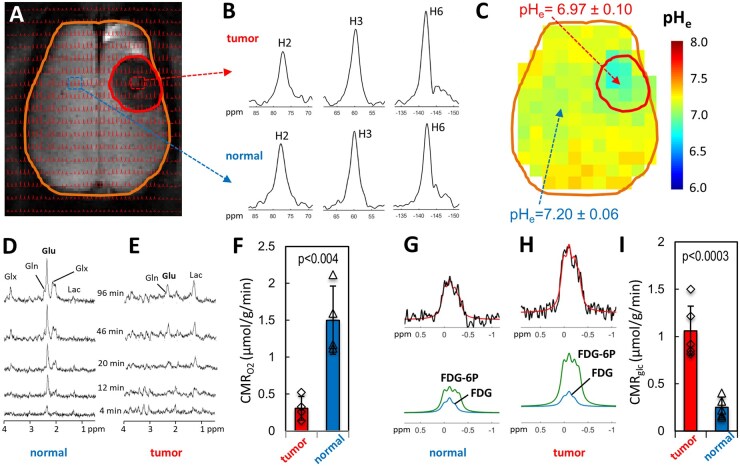
Cancer-initiated aerobic glycolysis (Warburg effect) is expressed by acidic extracellular pH (pH_e_) arising from uncoupling between consumption of oxygen (CMR_O2_) and glucose (CMR_glc_). (A) MRSI of rat brain, where the tumor was identified by hypointensity in the *T*_2_-weighted MRI (9L tumor is outlined within the right hemisphere, brain is outlined). The MRSI spectra in each voxel were overlayed on the *T*_2_-weighted MRI. The MRI hypointensity in the tumor is due to increased tumor vascular permeability allowing more TmDOTP^5-^ to enter the extracellular space compared to normal tissue, where TmDOTP^5-^ acts as a T_2_ MRI contrast agent. (B) Examples of MRSI signals from the H2, H3 and H6 protons of TmDOTP^5-^ inside tumor (top) and normal tissue (bottom) allowed the determination pH_e_ but also temperature (see Methods S2.1). (C) The corresponding pH_e_ map showing a lower pH_e_ = 6.97 ± 0.10 inside the tumor, compared to pH_e_ = 7.20 ± 0.06 outside the tumor. (D-F) Regional CMR_O2_ measured by localized POCE obtained during a steady-state [1,6-^13^C]-D-glucose infusion for 120 minutes. The POCE spectra showed ^13^C turnover from glucose at C1, C6 into glutamate (Glu) at C4, glutamine (Gln) at C4, mixture of glutamate and glutamine (Glx) at C3, and lactate (Lac) at C2, in normal tissue (D) and inside the tumor (E). The time-dependent incorporation of ^13^C label into the Glu C4 peak allowed calculation of CMR_O2_ (see Methods S2.2; Figure S1A). (F) The POCE measurements show that CMR_O2_ of normal tissue was 5-fold higher than CMR_O2_ inside the tumor (*P* < 0.004; *n* = 4 for each group). (G-I) Regional CMR_glc_ determined by ^19^F-MRSI during steady-state FDG infusion for 45 minutes. Time-dependent ^19^F-MRSI data showed conversion of blood FDG into tissue FDG and tissue FDG-6P in normal tissue (G) and inside the tumor (H), where the red line indicates the fit of the ^19^F signal to a sum of Gaussian line shapes representing FDG and FDG-6P signals. The time-dependent incorporation of ^19^F label into tissue FDG and FDG-6P allowed calculation of CMR_glc_ (see Methods S2.3; Figure S1B). (I) The ^19^F-MRSI results show that CMR_glc_ in normal tissue was about 4-fold lower than CMR_glc_ inside the tumor (*P* < 0.0003; *n* = 5). Individual data points in (F) and (I) are indicated by open diamond symbols for tumors and open triangles for normal tissue.

Figure 1 shows cancer-initiated AG (or Warburg effect), which is expressed by acidic pH_e_ due to uncoupling between CMR_O2_ and CMR_glc_ in the TME. Definition of tumor boundary remains a clinical challenge due to infiltrative nature of cancer cells growth. For consistency, throughout the paper we used the hypointensity in the *T*_2_-weighted MRI to differentiate the tumor from normal tissue (Figure 1A), approach also used clinically. For experiments not involving infusion of a contrast agent like TmDOTP^5-^ or TmDOTMA^-^ (eg, Figure S4A), we used either the *T*_2_ or the *T*_1_-weighted MRI for tumor delineation (depending on which provided the better contrast), since less than 10% difference in the tumor area was observed when comparing these 2 imaging methods.

The MRSI data from BIRDS were overlaid on the MRI (Figure 1A) to allow spatial localization of H2, H3 and H6 signals from TmDOTP^5-^ (Figure 1B), which were used to calculate the pH_e_ in each voxel (Methods S2.1; Equation (S2.1)). The pH_e_ map (Figure 1C) indicates an average pH_e_ value of 6.97 ± 0.10 in the 9L tumor, compared to a normal tissue pH_e_ value of 7.20 ± 0.06.

To compare CMR_O2_ in 9L tumors with that in normal tissue, we used localized ^1^H-[^13^C] MRS or POCE obtained during steady-state [1,6-^13^C]-D-glucose infusion. The POCE spectra obtained from a voxel positioned either in normal tissue (Figure 1D) or inside the tumor (Figure 1E) showed ^13^C turnover from ^13^C-labeled glucose at positions C1 and C6 into glutamate at C4, glutamine at C4, a sum of glutamate and glutamine at C3, and lactate at C2. A simple approach can be employed to determine CMR_O2_ by measuring the initial slope of the time-dependent incorporation of ^13^C label into the glutamate C4 peak (Figure S1A), where the initial slope is proportional to CMR_O2_ (Methods S2.2; Equation (S2.2)).^34^ The results show that the average initial slope in normal tissue was significantly higher (5-fold, *P* < .004; *n* = 4) than that measured inside the tumor. Assuming a CMR_O2_ value of 1.5 µmol/g/min in normal brain tissue for α-chloralose anesthetized rats,^34^ we calculated an average CMR_O2_ value of 0.3 ± 0.2 µmol/g/min inside the 9L tumors (Figure 1F).

CMR_glc_ was measured with ^19^F-MRSI during FDG infusion. The ^19^F-MRSI data showed the conversion of blood FDG into tissue FDG (FDG_t_) and phosphorylated FDG (FDG-6P) in normal brain (Figure 1G) and inside the 9L tumor (Figure 1H). The ^19^F signal was fitted to a sum of Gaussian line shapes (red line) representing total FDG (blue line) and FDG-6P (green line) signals. The incorporation of ^19^F label into FDG_t_ and FDG-6P allows calculation of CMR_glc_ (Methods S2.3; Equation (S2.3); Figure S1B). The ^19^F-MRSI measurements show that the average CMR_glc_ in normal tissue (0.25 ± 0.11 µmol/g/min) was about 4-fold lower than the average CMR_glc_ inside the tumor (1.06 ± 0.26 µmol/g/min; *P* < .0003, *n* = 5; Figure 1I).

**Figure 2.**
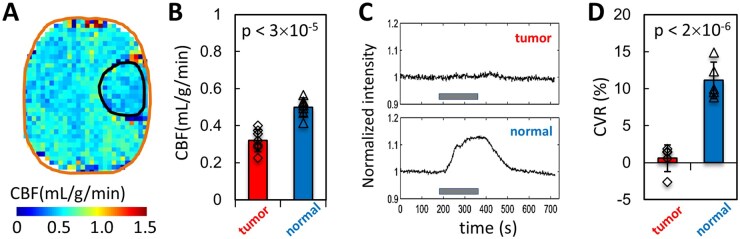
Cancer-initiated endothelial dysfunction is described by measurements of cerebral blood flow (CBF) and cerebrovascular reactivity (CVR) imaging. (A) The CBF map, based on 2 separate PASL measurements with inversion recovery TIR values of 2.1 s and 2.6 s, respectively, accounting for significant *T*_1_ differences between tumor and normal tissues (Figure S2). (B) The results show lower CBF in tumor (∼60%) compared to normal tissue at a high significance level (*P* < 0.00003; *n* = 7). (C) The CVR of blood vessels inside tumor (top) and in normal tissue (bottom) was measured by relative fMRI signal changes induced by vasodilation associated with arterial pCO_2_ rise. (D) The CVR responses during hypercapnia challenge were significantly higher in normal tissue compared to tumors (*P* < 0.000002; *n* = 5), indicating that the immature blood vessels inside the tumor were either maximally dilated and/or were not sensitive to rise in arterial pCO_2_. Individual data points in (B) and (D) are indicated by open diamond symbols for tumors and open triangles for normal tissue.

Figure 2 shows cancer-initiated ED by CBF and CVR ­imaging. CBF was obtained in rat brains with 9L tumors (Figure 2A). Because of *T*_1_ differences between tumor and normal tissue (Figure S2A), 2 datasets were acquired using 2 PASL acquisitions with TIRs of 2.1s for normal (Figure S2B) and 2.6s for tumor (Figure S2C) tissues and then combined to generate the final CBF map (Figure S2D). The results show that the average CBF (Methods S2.4; Equation (S2.4)) is ∼60% lower in 9L tumors (0.32 ± 0.06 mL/g/min) compared to normal tissue (0.50 ± 0.05 mL/g/min) at a high significance level (*P* < 0.00003; *n* = 7; Figure 2B). To probe CVR, which measures the capacity of blood vessels to dilate, we employed a mild hypercapnia challenge induced by an increase in arterial pCO_2_ (Methods S2.4). The *T*_2_*-weighted MRI signal change induced by vasodilation associated with arterial pCO_2_ rise was measured inside tumor (Figure 2C, top) and in normal tissue (Figure 2C, bottom). The amplitude of CVR responses during hypercapnia challenge (Figure 2D) were significantly higher in normal tissue compared to tumors (*P* < 0.000002; *n* = 5), indicating that the immature blood vessels inside the TME were either maximally dilated and/or were not sensitive to the rise in arterial pCO_2_.

**Figure 3.**
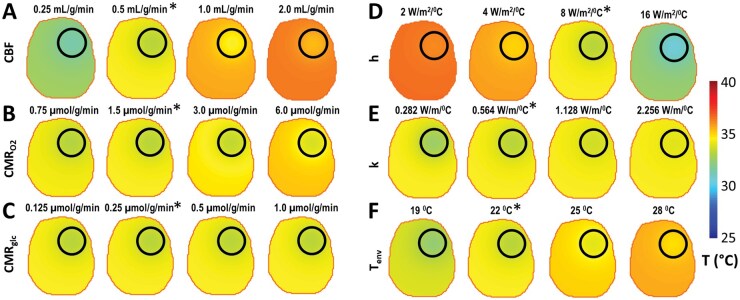
Simulations of brain temperature (T) maps using a 2D steady-state heat transfer model. The simulated T maps were calculated using the modified Pennes bioheat equation, where physiological parameters (CBF, CMR_O2_, and CMR_glc_) and biophysical parameters such as heat transfer coefficient (*h*), thermal conductivity (*k*), and the environmental temperature (*T_env_*) influenced the distribution of T in the brain (see Methods; Equations (2)). The simulated T maps represent a horizontal section of a rat brain, which includes a spherical tumor (5 mm diameter) in the right fronto-parietal region (tumor = dark outline, brain = light outline). To study how modifying a parameter value affects the tumor temperature (*T_t_*), the normal brain temperature (*T_n_*), and the Δ*T* gradient between normal and tumor tissue, we calculated the *T* distribution by individually changing the absolute values of each of CBF, CMR_O2_, CMR_glc_, *h*, *k* and *T*_env_ one parameter at a time, while keeping the rest of the parameters values constant and equal to those measured in this study (Figures 1 and 2). Note that for all simulations, the ratios between the tumor and normal tissue parameter values were kept constant and equal to those measured in this study (Table S2), except for *k* and *T_env_* values, which were considered the same for the voxels inside and outside the tumor. The difference in the *h* value between normal and tumor tissue was based on the difference in cellular density in gliomas compared to cortical tissue.^56^ See Figure S3 for simulations where the tumor value for each parameter was changed from the measured value, while the normal brain tissue parameters values were kept constant and equal to the measured values. The absolute value for the normal tissue for the parameter modified in each case is indicated above each map. The simulated *T* maps that are calculated using the measured CBF, CMR_O2_, CMR_glc_ and *T_env_* (Figures 4-6) are marked with *. (A) When normal CBF was increased from 0.25 to 2.0 mL/g/min, both *T_n_* and *T_t_* increased significantly by 3.71 and 3.85 °C, respectively, while Δ*T* decreased slightly by 0.14 °C. (B) When normal CMR_O2_ was increased from 0.75 to 6.0 μmol/g/min, both *T_n_* and *T_t_* increased by 0.86 and 0.74 °C, respectively, while Δ*T* increased only by 0.12 °C. (C) When normal CMR_glc_ was increased from 0.125 to 1.0 μmol/g/min, *T_n_*, *T_t_*, and Δ*T* did not change significantly. (D) When normal *h* was increased from 2 to 16 J/s/m^2^/g/°C, both *T_n_* and *T_t_* decreased significantly by 4.19 and 5.12 °C, respectively, while Δ*T* increased significantly by 0.93 °C. (E) When normal *k* was increased from 0.282 to 2.256 J/s/m/°C, *T_n_* was not significantly affected, while *T_t_* increased by 1.08 °C and Δ*T* decreased significantly by 0.98 °C. (F) When *T_env_* was increased from 19 to 28 °C, both *T_n_* and *T_t_* increased by 1.80 and 2.26 °C, respectively, while Δ*T* was reduced by 0.46 °C. All *T_n_*, *T_t_*, and Δ*T* values obtained from these simulations are included in Table S3.

A 3D steady-state heat transfer model based on a modified Pennes bioheat model^13^ was used to calculate the temperature (T) distribution in a horizontal section of a rat brain with a spherical tumor of 5 mm diameter ­(Figure 3, dark outline) located in the right frontoparietal region. In these simulations, the total heat was described using 4 heat components (Equation (2)). The parameters for these simulations used measured values and physiological constants (Table S1). For all simulations shown in Figure 3, the ratios between the tumor and normal tissue parameters were kept constant as determined from this study (Table S2). The thermal conductivity *k* = 0.56 J/s/m/°C, the extracranial environmental temperature *T_env_* = 22 °C (measured by an optical temperature probe positioned next to animal’s head inside the magnet bore) and the arterial temperature *T_art_* = 37 °C (considered equal to core temperature and measured by a rectal optical probe) were assumed the same inside and outside the tumor. The heat transfer coefficients used for the tumor and normal tissue were *h_t_* = 20 J/s/m^2^/g/°C and *h_n_* = 8 J/s/m^2^/g/°C, respectively (Tables S1 and S2). While *h_n_* has been previously measured^32^, we estimated *h_t_* based on cellular density differences for tumor and normal tissue as measured by ADC values (Figure S4), where 30% change in ADC corresponds to 250% change in cellular density.^35^ First, we calculated the T distribution using the measured CBF, CMR_O2_, and CMR_glc_ values. The results indicate an average tumor temperature (*T_t_*) of 33.45 ± 0.14 °C and an average normal brain temperature (*T_n_*) of 34.25 ± 0.20 °C (Figure 3, maps marked with *; Table S3). Thus, the temperature gradient (Δ*T* = *T_n_* — *T_t_*) between normal and tumor tissue was 0.8 °C (Table S3).

To study how modifying a parameter affects the T ­distribution, we individually changed the values of CBF, CMR_O2_, CMR_glc_, *h*, *k*, and *T_env_*, while keeping the rest of the parame­ters constant and equal to those measured in this study (Figure 3). Increasing normal CBF from 0.25 to 2.0 mL/g/min resulted in an increase of both *T_n_* and *T_t_* by 3.71 and 3.85 °C, respectively, while Δ*T* decreased by 0.14°C (Figure 3A). Increasing normal CMR_O2_ from 0.75 to 6.0 μmol/g/min resulted in an increase of both *T_n_* and *T_t_* by 0.86 and 0.74 °C, respectively, with Δ*T* increased by 0.12 °C (Figure 3B). When normal CMR_glc_ was increased from 0.125 to 1.0 μmol/g/min, values of *T_n_*, *T_t_*, and Δ*T* did not change significantly (Figure 3C). When normal *h_n_* was increased from 2 to 16 J/s/m^2^/°C, both *T_n_* and *T_t_* decreased significantly by 4.19 and 5.12 °C, respectively, while Δ*T* increased by 0.93 °C (Figure 3D). Increasing normal *k* from 0.282 to 2.256 J/s/m/°C resulted in an increase of *T_t_* by 1.08 °C, while *T_n_* increased only by 0.11 °C and Δ*T* decreased by 0.98 °C (Figure 3E). When *T_env_* was increased from 19 to 28 °C, both *T_n_* and *T_t_* increased by 1.80 and 2.26 °C, respectively, while Δ*T* was reduced by 0.46 °C (Figure 3F). All *T_n_*, *T_t_*, and Δ*T* values from simulations are shown in Table S3.

Similar simulations were run to determine how the T distribution is affected by individually changing the tumor/normal ratio for each parameter while keeping all normal tissue and the rest of tumor parameter values constant and equal to the measured values (Figure S3). Similar to previous simulations (Figure 3), *k* and *T_env_* were assumed to be the same inside and outside the tumor. The effect of their variation was already shown in Figure 3E-F. When CBF_t_/CBF_n_ was changed from 0.06 to 6, both *T_n_* and *T_t_* increased by 0.55 and 2.24 °C, respectively, while Δ*T* decreased by 1.69 °C (Figure S3A). When CMR_O2,t_/CMR_O2,n_, or CMR_glc,t_/CMR_glc,n_ were increased by 2 orders of magnitude, *T_n_*, *T_t_* and Δ*T* did not change significantly (Figure S3B-C). However, when *h_t_*/*h_n_* was increased from 0.25 to 25, both *T_n_* and *T_t_* decreased significantly by 1.72 and 6.90 °C, respectively, while Δ*T* increased significantly by 5.18 °C (Figure S3D). All *T_n_*, *T_t_*, and Δ*T* values obtained from these simulations are given in Table S4.

**Figure 4.**
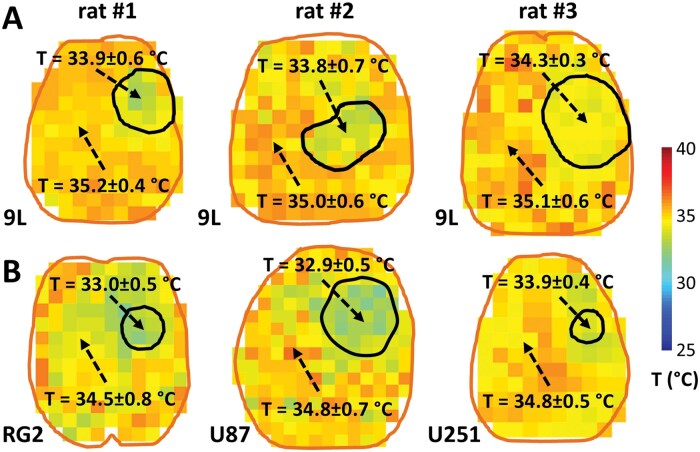
Measurements of brain temperature (*T*) by BIRDS using TmDOTP^5-^ in rat brain with various tumor types. (A) Examples of absolute *T* maps obtained with BIRDS (Methods S2.1; Equation (S2.1)) in 3 different rats bearing 9L tumors. The results indicate a lower *T* inside the tumor (*rat#1: T* = 33.9 ± 0.6 °C; *rat#2: T* = 33.8 ± 0.7 °C; *rat#3: T* = 34.3 ± 0.3 °C) compared to the average *T* outside the tumor (*rat#1: T* = 35.2 ± 0.4 °C; *rat#2: T* = 35.0 ± 0.6 °C; *rat#3: T* = 35.1 ± 0.6 °C). (B) Examples of absolute *T* maps with BIRDS in 3 different rats bearing 3 different types of tumors, RG2 (left), U87 (middle), and U251 (right). The results also show a lower *T* inside the tumor (*RG2: T* = 33.0 ± 0.5 °C; *U87: T* = 32.9 ± 0.5 °C; *U251: T* = 33.9 ± 0.4 °C) compared to the average *T* outside the tumor (*RG2: T* = 34.5 ± 0.8 °C; *U87: T* = 34.8 ± 0.7 °C; *U251: T* = 34.8 ± 0.5 °C).

**Figure 5.**
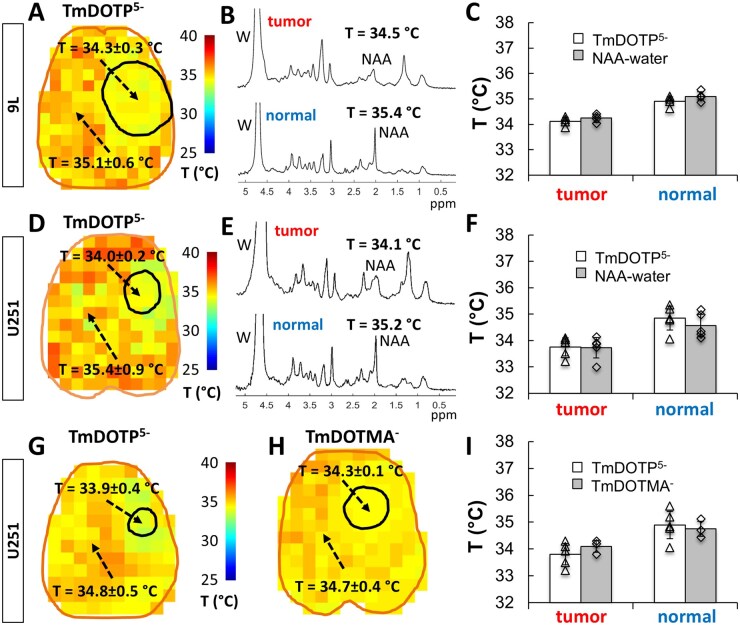
Validation of temperature (*T*) measured by BIRDS with TmDOTP^5-^, where in (A) to (F) the dual measurements were made in the same rat. Absolute *T* measurements were obtained in rats bearing 9L tumors with (A) BIRDS with TmDOTP^5-^ and (B) single voxel ^1^H-MRS with NAA-water either inside the tumor (top) or in normal tissue (bottom). The *T* inside the tumor was 34.3 ± 0.3 °C by BIRDS (averaged over all tumor voxels) and 34.5 °C by NAA-water chemical shift difference, while *T* outside the tumor was 35.1 ± 0.6 °C by BIRDS and 35.4 °C by NAA-water. (C) Average *T* values for 9L tumors measured by BIRDS with TmDOTP^5-^ (open bars) and ^1^H-MRS with NAA-water (filled bars) across a group of rats (*n* = 4) showed that the *T* values measured by both methods inside the tumor were lower than values measured in normal tissue (*P* < 0.008). We found a good agreement between *T* values measured by NAA-water and BIRDS, both inside and outside tumors (*P* > 0.31). Similar measurements were obtained in rats bearing U251 tumors: (D) BIRDS with TmDOTP^5-^ and (E) single voxel ^1^H-MRS with NAA-water either inside the tumor (top) or in normal tissue (bottom). The *T* inside the tumor was 34.0 ± 0.2 °C by BIRDS (averaged over all tumor voxels) and 34.1 °C by NAA-water chemical shift difference, while *T* outside the tumor was 35.4 ± 0.9 °C by BIRDS and 35.2 °C by NAA-water. (F) Average *T* values for U251 tumors measured by ^1^H-MRS with NAA-water (filled bars) and BIRDS with TmDOTP^5-^ (open bars) across a group of rats (*n* = 5) show also that *T* measured by both methods inside the tumor were lower than those in normal tissue (*P* < 0.012). Again, we found a good agreement between *T* with NAA-water and *T* with BIRDS, both inside and outside the U251 tumors (*P* > 0.30). The temperature difference between inside and outside the U251 tumors measured by BIRDS with TmDOTP^5-^ (G) was also confirmed using BIRDS with TmDOTMA^-^ (H). The *T* inside the tumor was 33.9 ± 0.4 °C by BIRDS with TmDOTP^5-^ and 34.3 ± 0.1 °C by BIRDS with TmDOTMA^-^, while *T* outside the tumor was 34.8 ± 0.5 °C by BIRDS with TmDOTP^5-^ and 34.7 ± 0.4 °C by BIRDS with TmDOTMA^-^. (I) Average *T* values for U251 tumors measured by BIRDS with TmDOTP^5-^ (open bars, *n* = 5) and BIRDS with TmDOTMA^-^ (filled bars, *n* = 3) show also that the *T* values measured by both methods inside the tumor were lower than those measured outside (*P* < 0.007). We found a good agreement between *T* measured using BIRDS with TmDOTP^5-^ and BIRDS with TmDOTMA^-^, both inside and outside tumors (*P* > 0.28). The *T* maps for BIRDS with TmDOTP^5-^ were obtained from the ^1^H chemical shifts of H2, H3, and H6 TmDOTP^5-^ protons (see Methods). The *T* measured by single voxel ^1^H-MRS with NAA-water was calculated from the ^1^H chemical shift difference between water and NAA signals (see Methods; Equation (10)). The *T* maps for BIRDS with TmDOTMA^-^ were obtained from the ^1^H chemical shift of the methyl group of TmDOTMA^-^ (see Methods S2.1; Equation (2.1c)). Individual data points in (C), (F), and (I) are indicated by open triangles for BIRDS with TmDOTP^5-^ and open diamond symbols for NAA-water and BIRDS with TmDOTMA^-^.

To explore the temperature gradient (Δ*T*) between normal and tumor tissue, we used BIRDS with TmDOTP^5-^ to measure the brain temperature in 4 different tumor cell lines, 9L, RG2, U87, and U251 (Figure 4). For most of these measurements, the Δ*T* was positive, indicating that the tumor temperature was cooler than normal brain tissue. For example, for the 6 rats shown in Figure 4, Δ*T* was between 0.8 and 1.9 °C. Significant differences were not observed when the average measured tumor temperature was calculated using a larger tumor boundary (20%-30% larger diameter) compared to that measured using the *T*_2_-weighted MRI. To validate Δ*T* measured by BIRDS with TmDOTP^5-^ (Methods S2.1, Equation (S2.1a)) which were measured simultaneously with the pH_e_ (Methods S2.1, Equation (S2.1b)), prior to TmDOTP^5-^ infusion we measured *T_n_*, *T_t_*, and Δ*T* using single voxel ^1^H-MRS in rats with either 9L tumors (Figure 5A-C) or U251 tumors ­(Figure 5D-F). T measurements with ^1^H-MRS are based on NAA-water chemical shift difference (Methods S2.1, Equation (S2.1d)). Independent measurements were obtained inside tumors (top spectra of Figure 5B and E) and in the normal brain tissue (bottom spectra of Figure 5B and E). For the 9L tumor example, we found *T_n_*  = 35.1 ± 0.6 °C, *T_t_* = 34.3 ± 0.3 °C, and Δ*T*  = 0.8°C by BIRDS with TmDOTP^5-^ (Figure 5A), while *T_n_* = 35.4 °C, *T_t_* = 34.5 °C, and Δ*T* = 0.9 °C were measured by ^1^H-MRS with NAA-water (Figure 5B). Group results for 9L tumors (Figure 5C, *n* = 4) indicate the same trend: 9L tumors have a lower temperature (34.1 ± 0.2 °C by BIRDS, 34.3 ± 0.2 °C by ^1^H-MRS) compared to the surrounding normal tissue (34.9 ± 0.2 °C by BIRDS, 35.1 ± 0.2 °C by ^1^H-MRS). For the U251 tumor example, we found *T_n_* = 35.4 ± 0.9 °C, *T_t_* = 34.0 ± 0.2 °C, and Δ*T* = 1.4 °C by BIRDS with TmDOTP^5-^ (Figure 5D), while *T_n_* = 35.2 °C, *T_t_* = 34.1 °C, and Δ*T* = 1.1 °C were measured by ^1^H-MRS with NAA-water (Figure 5E). Group results for the U251 tumors (Figure 5F, *n* = 5) also indicate that tumors have a lower temperature (33.8 ± 0.3 °C by BIRDS, 33.7 ± 0.4 °C by ^1^H-MRS) compared to normal tissue (34.9 ± 0.5 °C by BIRDS, 34.6 ± 0.4 °C by ^1^H-MRS).

The T measurements by BIRDS with TmDOTP^5-^ in U251 tumors (*n* = 5) were further validated using BIRDS with TmDOTMA^-^ (Figure 5G-I), a method based on the temperature dependence of TmDOTMA^-^ methyl chemical shift ­(Methods S2.1, Equation (S2.1c)). Figure S5 shows a detailed example of temperature imaging using BIRDS with TmDOTMA^-^, including the 3D MRSI data (Figure S5B), examples of ^1^H spectra showing the methyl resonance from the U251 tumor and normal tissue (Figure S5C) and the corresponding T map (Figure S5D). Note that the T measurements by BIRDS with TmDOTMA^-^ were obtained in a separate group of animals (*n* = 3). The group results (Figure 5I) indicate that the U251 tumors have a lower temperature (33.8 ± 0.3 °C by BIRDS with TmDOTP^5-^, 34.1 ± 0.1 °C by BIRDS with TmDOTMA^-^) compared to normal tissue (34.9 ± 0.5 °C by BIRDS with TmDOTP^5-^, 34.8 ± 0.2 °C by BIRDS with TmDOTMA^-^).

**Figure 6.**
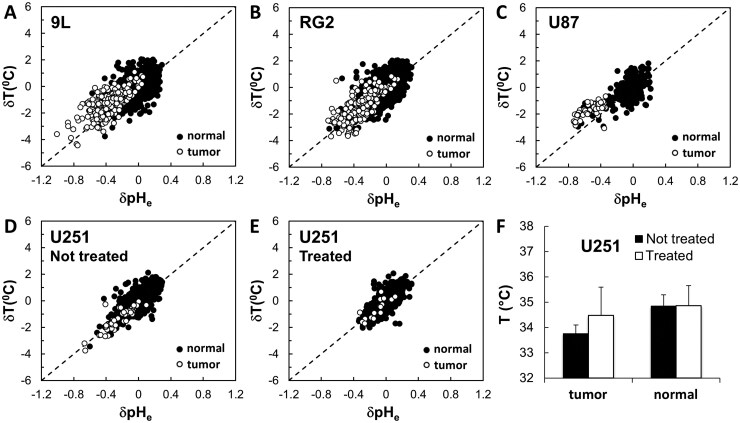
Temperature-pH_e_ correlation for 9L, RG2, U87, and U251 tumors. The pH_e_ and temperature deviations (δpH_e_ and δ*T*) from the average pH_e_ and *T* values (measured outside the tumor, see Methods; Equations (7) and (8)) were plotted against each other for: (A) 9L (*r_t_* = 0.69, *r_n_* = 0.64, *r_all_* = 0.74, *n* = 13); (B) RG2 (*r_t_* = 0.76, *r_n_* = 0.77, *r_all_* = 0.81, *n* = 15); (C) U87 (*r_t_* = 0.62, *r_n_* = 0.65, *r_all_* = 0.77, *n* = 2); (D) not treated U251 (*r_t_* = 0.89, *r_n_* = 0.74, *r_all_* = 0.79, *n* = 6); and (E) treated U251 (*r_t_* = 0.81, *r_n_* = 0.73, *r_all_* = 0.74, *n* = 5) tumors. A high T-pH_e_ correlation (*r* > 0.6) was observed both inside tumors and in normal tissue for all 4 different types of brain tumors investigated. Treatment of U251 tumors with Temozolomide (F) reduced the temperature gradient Δ*T* between normal tissue and tumor (Δ*T* = 0.4 °C) compared to not-treated animals (Δ*T* = 1.1 °C).

BIRDS with TmDOTP^5-^ allows simultaneous measurements of temperature (Methods S2.1, Equation (S2.1a)) and pH_e_ (Methods S2.1, Equation (S2.1b)).^23^^,^^24^ Our results indicate that both pH_e_ and T are lower in tumors compared to normal tissue (Figures 1C, 4, and 5). To investigate if there is a correlation between these 2 parameters, we plotted the pH_e_ and temperature deviations (δpH_e_ and δT) between tumor and normal tissue for 9L, RG2, and U87 tumors ­(Figure 6A-C), as well as treated and untreated U251 tumors (Figure 6D-E). A high T-pH_e_ correlation (*r* > 0.6) was observed both for tumors and normal tissue for 9L (*r_t_* = 0.69, *r_n_* = 0.64, *n* = 13), RG2 (*r_t_* = 0.76, *r_n_* = 0.77, *n* = 15), U87 (*r_t_* = 0.62, *r_n_* = 0.65, *n* = 2), untreated U251 (*r_t_* = 0.89, *r_n_* = 0.74, *n* = 6), and treated U251 (*r_t_* = 0.81, *r_n_* = 0.73, *n* = 5) tumors. The correlation improved (*r* > 0.7) for 9L, RG2, and U87 tumors when the tumor and normal tissue data were analyzed together (*r* = 0.74 for 9L; *r* = 0.81 for RG2; *r* = 0.77 for U87), while for untreated and treated U251 tumors, the correlation improved only when compared to normal tissue (*r* = 0.79 for untreated U251; *r* = 0.74 for treated U251). Interestingly, treatment of U251 tumors with Temozolomide (Figure 6F) reduced the Δ*T* between normal tissue and tumor (*T_n_* = 34.9 ± 0.8 °C, *T_t_* = 34.5 ± 1.1 °C, Δ*T* = 0.4 °C) compared to untreated rats (*T_n_* = 34.9 ± 0.5 °C, *T_t_* = 33.8 ± 0.3 °C, Δ*T* = 1.1 °C), although T-pH_e_ correlations were not altered (Figure 6D-E). A moderate correlation (0.1 < *r* < 0.5) was observed between ΔT and tumor diameter (Figure S6) for 9L (*r* = 0.32, *n* = 13), RG2 (*r* = 0.10, *n* = 15), U251 (*r* = 0.48, *n* = 6), and U87(*r*=not available, *n* = 2). The tumor diameter was estimated from *T*_2_-weighted MRI. For U87 tumors, *r*-value was not available.

## Discussion

In normal brain tissue, glucose oxidation contributes to most of the heat production, which if not efficiently cooled could result in a significant increase in brain temperature. High temperature (> 38 °C) has a negative impact on the central nervous system due to its vulnerability to hyperthermia causing irreversible damage or even death.^36^ Efficient and necessary heat removal is mainly achieved by controlling blood flow.^31^ However, in the TME there is an uncoupling between blood flow (40% CBF reduction) and glucose oxidation (4.2× higher CMR_glc_ and 4.8× lower CMR_O2_).^15^ Our hypothesis is that because of uncoupling between CMR_O2_ and CMR_glc_ (Equation (1a)) as well as CMR_O2_ and CBF (Equation (1b)) in the TME, the temperature control becomes deficient resulting in temperature differences between tumor and normal tissue. We observed CMR_O2_-CMR_glc_ coupling in normal tissue but significant CMR_O2_-CMR_glc_ uncoupling in the TME (Figure 1). While CBF was lower in tumors (Figure 2A), the difference was less pronounced (∼40%) than for CMR_O2_ and CMR_glc_ reductions in tumors (Figure 1). CVR measurements in the TME indicate a significant lack of blood vessel reactivity (Figure and D), suggesting that immature blood vessels were either maximally dilated and/or were not sensitive to arterial pCO_2_ increase. This lack of reactivity of tumor blood vessels has been observed by others.^37^

To test if uncoupling between CMR_O2_ vs CMR_glc_ (Equation (1a)) and CMR_O2_ vs CBF (Equation (1b)) can impact temperature differences between tumor and normal tissue, we used a heat transfer model based on modified Pennes bioheat equation at steady-state (Equation (2))^13^ to calculate the temperature distribution in rat brain with a spherical tumor of 5 mm diameter (Figure 3), previously used to model temperature distribution in neonatal head.^32^ Because temperature mapping with BIRDS was investigated in a single slice positioned close to the brain surface underneath the skull where the heat exchange with the extracranial environment occurs in a direction perpendicular to the slice, we chose to conduct our simulations in a 3D volume comprising a single 1 mm slice instead of involving the whole brain. To better emulate the *in vivo* temperature measurements with BIRDS, a perpendicular surface heat loss component (Equation (2d)) was included, but only for one side of the slice investigated, corresponding to heat loss through the skull. Thus, the simulations were conducted in a 3D manner where volumetric parameters were expressed in 3D units and heat exchange components were assumed over 2D surfaces. Choosing a superficial slice for temperature measurements was based on much higher signal-to-noise ratio (SNR) for BIRDS compared to deeper slices where SNR for BIRDS decreases significantly with greater distance from the surface RF coil. Using a volume coil for excitation/acquisition was not feasible due to limitations imposed by much higher RF power requirements for transmission and lower RF sensitivity for acquisition (lower SNR). Using the measured values of CBF, CMR_O2_, CMR_glc_, *T_env_*, and *T_art_* in rat brain with other parameters from literature (Tables S1 and S2), we calculated that temperature was higher in normal tissue (34.25 ± 0.20 °C) compared to the tumor (33.45 ± 0.14 °C), resulting in a temperature gradient (Δ*T*) of +0.8°C (Figure 3 and Table S3). These *in silico* Δ*T* results (Figure 3) are in excellent agreement with *in vivo* Δ*T* results (Figure 4).

To understand how various parameters affect the temperature distribution, we modified one parameter at a time, while keeping the ratio and the other parameters constant (Table S3). Although these variations extend beyond biologically relevant ranges, the results help us determine what parameters have significant effects on temperature. For some parameters (eg, CMR_O2_, CMR_glc_, and *k*) their changes impacted TME temperature by less than 1 °C, while for other parameters (eg, CBF, *h*, and *T_env_*) their changes affected TME temperature by more than 1 °C. Using these results one can also easily estimate the effect on temperature when the parameters are varied in a more biologically relevant range. The results indicate that increasing CBF results in a significant increase (>3 °C) in both *T_n_* and *T_t_* (Figure 3A) because more heat is delivered to the brain since the arterial temperature *T_art_* = 37 °C is higher than the brain temperature. However, changing CBF by almost one order of magnitude did not significantly affect Δ*T* which remained in the range 0.62-0.80 °C. Interestingly, the maximum Δ*T* (0.8 °C) was obtained for the experimentally measured parameters. Another parameter that significantly affected the brain temperature was the heat transfer coefficient *h*. Because there is no method that can provide a direct measure of *h* distribution and because quantifying *h* from ADC via cell density is not possible, we used the same *h* value for all normal voxels and a higher *h* value for all tumor voxels. This assumption was based on previous measurements indicating that cellular density in gliomas is higher than that of normal brain tissue,^33^ an assumption supported by our *in vivo* ADC measurements (Figure S4). Our simulations involving varying *h* value for tumor while keeping the rest of the parameters unchanged (Figure S3D) explore the possibility that various tumor types or the same tumor at different stages of tumor progression might have different cell densities, resulting in different temperatures. Increasing *h* by almost one order of magnitude resulted in a significant decrease (>4 °C) in both *T_n_* and *T_t_* (Figure 3D) because in this case the efficiency of heat removal increases with *h*. The significant increase in Δ*T* (0.93 °C) is likely due to the higher *h* value in tumors (Table S3). Smaller increases (<2.3 °C) in both *T_n_* and *T_t_* were observed when CMR_O2_ was increased 8-fold (Figure 3B) or *T_env_* was increased from 19 to 28 °C (Figure 3F). A CMR_O2_ increase results in a larger heat ­production and therefore an overall increase in brain temperature. An increase in the extracranial environment temperature *T_env_* results in a smaller amount of heat removed from the brain (due to a decrease in brain-air temperature difference) and therefore an overall increase in brain temperature. The Δ*T* decreased by ∼50% (from 0.96 to 0.49 °C) when *T_env_* increased from 19 to 28 °C and increased only slightly (0.13 °C) with 8-fold CMR_O2_ increase (Table S3). The increase in thermal conductivity *k* by almost an order of magnitude (Figure 3E) selectively and significantly increased the tumor temperature (>1 °C) while the brain temperature increased only slightly (0.11 °C). As a result, Δ*T* decreased from 1.25 to 0.27 °C with *k* (Table S3) consistent with an increased heat exchange between tumor and normal tissue at higher *k* values. Interestingly, an 8-fold increase in CMR_glc_ did not significantly affect *T_n_*, *T_t_*, or Δ*T* (Figure 3C and Table S3), suggesting that contribution of AG to heat production is minimal, due to the small value of glycolytic enthalpy Δ*H_glyc_* = 0.061 J/μmol compared to the enthalpy of oxidative phosphorylation Δ*H*_0_ = 0.47 J/μmol (Table S1).

To determine how differences in parameters between tumor and normal tissue affect the temperature distribution and Δ*T*, we ran simulations in which the tumor/normal ratio for each parameter was changed, while keeping all normal tissue and the rest of tumor parameters constant (Figure S3). Increasing CBF_t_ by 2 orders of magnitude resulted in a large increase in *T_t_* (2.24 °C), but a smaller increase in *T_n_* (0.55 °C), leading to a large decrease in Δ*T* by 1.69 °C (ie, from +1.12 to −0.57 °C). This result is consistent with previous measurements indicating that CBF efficiently controls brain temperature.^31^ Moreover, our simulations show that the CBF_t_/CBF_n_ ratio not only influences the magnitude of Δ*T*, but can also invert its sign, making the tumor temperature higher than normal brain tissue temperature (Figure S3A). In contrast, increasing CMR_O2,_  _*t*_/CMR_O2,_  _*n*_ or CMR_glc,_  _*T*_/CMR_glc,_  _*N*_ by 2 orders of magnitude did not significantly change *T_n_*, *T_t_*, or Δ*T* (Figure S3B-C and Table S4), suggesting that the tumor metabolism (either oxidative or glycolytic) is not the main means by which the brain controls its temperature. Interestingly, changes by 2 orders of magnitude in the ratio h_t_/h_n_ of heat transfer to the extracranial environment resulted in the largest decrease in tumor temperature (∼7 °C), while the normal brain temperature was decreased by less than 2 °C (Figure S3D). However, changes in *h* by 2 orders of magnitude are very unlikely in the brain which is well insulated by the skull and skin layers.

There are several MRI-based methods for brain temperature mapping, based on changes in water resonance ­frequency, its *T*_1_ relaxation time, ADC, or using temperature-sensitive MRI contrast agents.^38^ The most widely used method applied in tumors is based on ^1^H-MRS to measure changes in water resonance frequency relative to NAA.^25^ The other methods are less common for temperature measurements in tumors, as they are sensitive to changes in protein content, ionic concentration or cell density.^39^ A variety of DOTA-based agents have been used for molecular imaging with MRI using relaxation methods or *C*hemical *E*xchange *S*aturation *T*ransfer (CEST).^40^ The relaxation ­methods rely on the paramagnetic effect induced by the paramagnetic cations on inner sphere of water protons, whereas CEST methods detect the chemical exchange between the bulk water and paramagnetically shifted inner sphere bound water molecules, or between bulk water protons and exchangeable protons of the ligand (eg, -NH_x_ or -OH). While some paramagnetic CEST agents can also provide temperature measurements,^41^ their use has been limited by the high RF power needs. BIRDS using ^1^H-MRSI with similar paramagnetic contrast agents (eg, TmDOTP^5-^ or TmDOTMA^-^) can provide temperature measurements with high accuracy.^23^^,^^24^ Temperature measurements using BIRDS with TmDOTP^5-^ are based on high temperature sensitivity of H2 (−0.6 ppm/°C), H3 (−0.5 ppm/°C), and H6 (1.0 ppm/°C) proton chemical shifts.^24^ The redundant information provided by using 3 peaks with slightly different sensitivities allows increased measurement accuracy. The accuracy of temperature measurements also depends on the SNR of these peaks—higher SNR would reduce the errors in chemical shift measurements and thus decrease the uncertainty of temperature estimation.^23^ For SNR > 5 typical for BIRDS we estimate an error of 0.04 ppm (20 Hz at 11.7T) in chemical shift measurements, which corresponds to an uncertainty of 0.1 °C for temperature measurements.^23^ Thus, BIRDS with TmDOTP^5-^ can measure temperature with a much higher accuracy than the ^1^H-MRS with NAA-water method, which has a sensitivity of only 0.01 ppm/°C.^25^ Therefore, we employed BIRDS with TmDOTP^5-^ to investigate if tumors have a lower temperature than normal tissue, as suggested by our simulations. Indeed, this result was confirmed in different animals with the same tumor type (9L; Figure 4A), and in animals with different tumor lines (RG2, U87, and U251; Figure 4B). The Δ*T* results were similar (∼1 °C lower temperature in tumors) in all cell lines investigated suggesting that Δ*T* is independent of tumor type.

Temperature measurements using BIRDS with TmDOTP^5-^ were validated using 2 other methods, ^1^H-MRS with NAA-water (Figure 5B and E) and BIRDS with TmDOTMA^-^ (Figures 5H-I and S5B). Due to its lower temperature sensitivity (albeit its relatively high SNR), we estimate an uncertainty of 0.4 °C for temperature measurements using the ^1^H-MRS with NAA-water method. In contrast, the BIRDS with TmDOTMA^-^ method based on measuring the chemical shift of a methyl group with ∼5-fold higher SNR than that of TmDOTP^5-^ protons^23^ provides temperature measurements with high accuracy. For SNR > 5 typical for BIRDS with TmDOTMA^-^ we estimate an error of 0.03 ppm (15 Hz at 11.7T) in chemical shift measurements, which corresponds to an uncertainty of 0.05 °C in temperature measurements.^23^ Both ^1^H-MRS with NAA-water and BIRDS with TmDOTMA^-^ confirmed the BIRDS with TmDOTP^5-^ temperature measurements in normal tissue, as well as in tumors (Figure 5C, F, and I). For all these measurements the tumor temperature was lower than that of normal tissue. In 9L tumors, the temperature gradient was Δ*T*  = 0.79 °C measured using BIRDS with TmDOTP^5-^ and Δ*T* = 0.84 °C by ^1^H-MRS (Figure 5C), while in U251 tumors, Δ*T* = 1.09 °C measured by BIRDS with TmDOTP^5-^, Δ*T* = 0.85 °C by ^1^H-MRS, and Δ*T* = 0.72 °C by ^1^H-MRS (Figure 5F and I). Because the contrast agents used for BIRDS are located exclusively outside the cells, the measurements by BIRDS (using either TmDOTP^5-^ or TmDOTMA^-^) reflect the temperature of the extracellular space. However, temperature measurements using ^1^H-MRS of NAA and water reflect predominantly the intracellular space. The agreement between temperature measurements with BIRDS and ^1^H-MRS indicates a minimal temperature gradient between intracellular and extracellular milieus (ie, equilibrium temperature was reached).

Temperature measurements using infrared imaging show that human brain tumors could be either cooler or warmer than the surrounding tissue.^42^ The temperature values measured in normal brain in the current work (in the range of 34-36 °C) are in good agreement with previous thermocouple measurements in anesthetized rat brain.^7^ A higher mitochondrial temperature (by 6-10 °C) was observed when the respiratory chain was fully functional using a temperature-sensitive fluorescent probe that targets mitochondria.^43^ ­However, these measurements were questioned by other studies that show a degradation of respiratory complexes at temperatures above 43 °C.^44^ Our simulations indicate that the brain temperature depends on the interplay of several factors (eg, CBF, CMR_O2_, CMR_glc_, *h*, *k*, and *T_env_*) which can affect the temperature gradient between tumor and normal tissue. Temperature differences between tumor and normal tissue were reported in the literature in benign and malignant lung tumors,^21^ urinary bladder tumors,^45^ or breast tumors.^46^ Based on these results, temperature might be included in the long list of physiological parameters that are significantly different in tumors compared to normal tissue, for example, pH_e_, CMR_O2_, CMR_glc_, CBF, ADC, and also sodium levels.^47^

BIRDS with TmDOTP^5-^ has been previously used to measure the intratumoral-peritumoral pH_e_ gradient in 9L and RG2 tumors.^16^ Because BIRDS can provide simultaneous measurements of temperature and pH_e_, and since our results indicate that both are lower in tumors, we investigated the potential correlation between these physiological parameters in different tumor cell lines (Figure 6). For this comparison we treated each voxel as independent measurement, pooled across subjects from within each tumor type and not from within each subject separately. The results indicate that a strong T-pH_e_ correlation (*r* > 0.6) exists in all cell lines when tumor and normal tissue are investigated separately. Moreover, the T-pH_e_ correlation improves (*r* > 0.7) for the 9L, RG2, and U87 cell lines when the tumor and normal tissue are analyzed together. However, for the U251 cell line, the T-pH_e_ correlation when considering together the tumor and normal tissue data increased when compared to normal tissue data but decreased when compared to tumor data. This discrepancy between U251 and the other 3 cell lines suggest that the U251 tumors possess phenotype differences which might be responsible for an even higher T-pH_e_ correlation (*r* > 0.8). It is important to note that the T-pH_e_ correlation observed inside and outside the TME is due to the presence of regions with lower temperature and pH_e_ beyond the tumor margins exclusively defined by MRI (Figure 1A), regions which potentially correspond to areas of tumor cell infiltration into normal tissue. Evidence suggests that a low temperature is preferred for tumor growth, either in the tumor core^48^ or systemically.^49^ While there is no physiochemical reason for the existence of a T-pH_e_ correlation, we propose that this arises due to a correlation between upregulated AG in the TME asiring from CMR_O2_-CMR_glc_ uncoupling (to acidify pH_e_) and ED in the TME due to CMR_O2_-CBF uncoupling (to lower temperature). Thus, reduction in the temperature gradient Δ*T* (Figure 6F) as well as pH_e_ gradient^17^ for treated tumors suggests that either temperature or pH_e_ can be used as biomarkers for therapeutic outcome.

Previous studies suggest that the temperature has an important role in regulating the immune response and that immunotherapy combined with thermal therapy may improve therapeutic outcome.^50^ The TME responds to ­hyperthermia through temperature-sensitive checkpoints that regulate tumor metabolism, perfusion, inflammatory cytokine expression, lymphocyte trafficking, as well as innate and adaptive immune functions.^50^ While temperature is rarely included in TME literature,^50^ our observation regarding lower temperature in tumors potentially impacts future therapeutic directions because both cooler and acidic conditions enhance tumor growth.^51^^,^^52^ Mild hyperthermia (39-43 °C) also boosts chemotherapy by increasing vascular permeability to drugs and interferes with DNA repair pathways.^53^ In this context, the number of studies regarding the importance of temperature regulation in cancer treatment is limited, and additional pre-clinical and clinical studies investigating the potential improvement of immunotherapies, radiotherapies or chemotherapies when combined with thermal therapy are needed.^54^ While acidic pH_e_ improves hyperthermia treatment of tumors,^55^ it is unknown to what degree pH_e_ is related to temperature, within and beyond the tumor core. However, a limitation of our study is that CBF, CMR_O2_, CMR_glc_ and temperature were measured in separate groups of animals, because these measurements require long acquisitions. In the future, the use of cryogenic RF coils to increase SNR and interleaved acquisitions using hybrid RF coils could potentially reduce the duration of these scans to allow simultaneous measurements. Temperature measurements in tumors from other organs (eg, liver, pancreas) should be obtained in the future to establish if a lower tumor temperature is a property specific only to gliomas or a general feature of all tumors.

In conclusion, cancer cell growth in GBM is orchestrated by a complex interplay between AG and ED, where AG promotes tumor invasion and suppresses immune response with extracellular acidification and ED obstructs perfusion to stimulate hypoxia because of leaky blood vessels. Therefore, we posit that temperature reflects combined effects of AG and ED. This study tested the complex interplay of how metabolism-driven heat generation and perfusion-driven heat removal leads to a cooler TME which was confirmed by various thermal imaging methods and simulations. The combined observations of our study suggest that TME is more glycolytic, acidic, hypoperfused, and hypothermic compared to its neighboring normal milieu. Since temperature was correlated with pH_e_ inside and outside the TME for untreated and treated rats, we propose that thermal mapping may represent cancer hallmarks (of AG and ED) for early GBM detection. Future studies with BIRDS can reveal if systemic cooling can enhance the tumor growth rates of gliomas.

## Supplementary Material

vdag007_Supplementary_Data

## Data Availability

The data generated in this study are available upon request from the corresponding author.
