# Supplementary material for: High-resolution thermal imaging to delineate effects of cancer-induced aerobic glycolysis and endothelial dysfunction
Source: Neurooncol Adv. 2026 Jan 18;8(1):vdag007. doi: 10.1093/noajnl/vdag007 (PMC13168813; doi:10.1093/noajnl/vdag007)
Supplement: vdag007_Supplementary_Data [file vdag007_supplementary_data.zip › 19-Feb-2026_023515_Supplementary_material.docx]

# **Supplementary Materials for**

**High-resolution thermal imaging to delineate effects of cancer-induced aerobic glycolysis and endothelial dysfunction**

Daniel Coman^1,2^, Peter Herman^1^, Jyotsna U. Rao^1^, Jelena Mihailovic^1^, Yuegao Huang^1^, Gen Kaneko^1^, Fahmeed Hyder^1,2^

^1^Yale University, Department of Radiology & Biomedical Imaging, New Haven, CT 06510, USA

^2^Yale University, Department of Biomedical Engineering, New Haven, CT 06510, USA

***Running title:*** Thermal mapping of metabolic-vascular changes in tumors

***Corresponding authors:***

Daniel Coman <daniel.coman@yale.edu>, 300 Cedar Street, N136 TAC, New Haven, CT 06520, *Phone: (203) 737-8066*

Fahmeed Hyder <fahmeed.hyder@yale.edu>, 300 Cedar Street, N143 TAC, New Haven, CT 06520 *Phone: (203) 785-6205*

## **Methods S1 - Tumor models, tumor implantation, and tumor treatment**

We used 9L, RG2, U87, and U251 lines in rat brain. The malignant 9L is a rat-derived gliosarcoma model that develops in Fisher 344 rats and closely resembles the high-grade malignant brain tumors in humans**^1^**. The malignant RG2 is a rat-derived glioma model that also develops in Fisher 344 rats**^2^**. The U87 and U251 are both human-derived glioma models that grow in athymic/nude rats**^3,4^**. All tumor cells were procured from the American Type Culture Collections (ATCC, Manassas, VA, USA). The cells were cultured in a 75 cm^3^ culture flasks in an incubator with 5% CO_2_ atmosphere at 37°C. The culture medium used was the Dulbecco's modified Eagle's medium (DMEM) supplemented with 10% heat-inactivated fetal bovine serum and 1% penicillin-streptomycin. 9L, RG2, and U87 cells were cultured in high glucose (4.5 g/L) DMEM, while U251 cells were cultured in low glucose (1.5 g/L) DMEM. Only cells at a low passage number (<20) were utilized. Prior to intracranial injection, the cells were harvested at high confluence (>80%) and suspended in serum-free DMEM.

All animal protocols were approved by the Institutional Animal Care and Use Committee at Yale University. Adult Fischer 344 rats (220-280 g; n = 48) and athymic nude rats (200–250 g; n = 16), were obtained from Yale University vendors and maintained according to approved animal care protocols. Animals were kept in temperature-controlled and humidity-controlled rooms with food and water available ad libitum for at least two weeks prior to magnetic resonance scans. We used 33 Fisher rats for 9L tumors and 15 Fisher rats for RG2 tumors for a total of 48 Fisher rats. The 9L rats (n=33) were used as follows: 13 for BIRDS with TmDOTP^5-^, 4 for CMR_O2_ with ^1^H-[^13^C] MRS, 5 for CMR_glc_ with ^19^F-MRSI, 7 for CBF and 4 for CVR. In one 9L rat two CVR datasets were obtained, increasing the total number of CVR datasets to 5. Temperature measurements with NAA/water were obtained in 4 of the 9L rats used for BIRDS with TmDOTP^5-^. The RG2 rats (n=15) were used only for BIRDS with TmDOTP^5-^. 14 athymic nude rats were implemented with U251 tumors; 6 untreated and 5 treated were used for BIRDS with TmDOTP^5-^ and 3 for BIRDS with TmDOTMA^-^. ADC and T measurements with NAA/water were obtained in 5 of the untreated U251 rats used for BIRDS with TmDOTP^5-^. 2 athymic nude rats were implemented with U87 tumors and used for BIRDS with TmDOTP^5-^.

For intracranial injection of cells, the rats were anesthetized (2-3% isoflurane) and positioned in a stereotaxic instrument. The cells at high confluence were harvested, washed, and suspended in DMEM. Intrathalamic injections were made with a 10 µL Hamilton syringe, fitted with a 26-gauge beveled needle, into the right striatum at 3 mm to the right of bregma and 3 mm below dura. Further, a volume (5μL) of cell suspension (9L = 2x10^5^ cells, RG2 = 2500 cells, U87 = 2x10^5^ cells, U251 = 5x10^5^ cells) was injected over the course of 5 minutes and the needle was left in place for an additional 5 minutes before it was slowly withdrawn. After injection the scalp was closed, treated with antibiotic, and non-steroidal anti-inflammatory drug (meloxicam, 1 mg/kg) was injected subcutaneously to prevent pain and inflammation. For pain management, carprofen (5 mg/kg) was administered subcutaneously during the procedure, and this treatment continued for 48 hours after the surgery to provide analgesia. Rats were monitored daily for signs of weight loss or development of neurological symptoms. Tumors were allowed to grow for a period between 14 to 26 days to reach a size between 1.6 mm and 7.8 mm when scanned (**Fig. S6**). In a few animals (n=5) with U251 tumors starting at 12 days post tumor injection, temozolomide was orally administered daily (40 mg/kg) in 2 cycles of 4 days each with a gap of 2 days between cycles.

## **Methods S2 - Details of temperature, pH_e_, CMR_O2_, CMR_glc_, CBF, CVR, and ADC measurements**

## S2.1 - Temperature and pH_e_ with Biosensor Imaging of Redundant Deviation in Shifts (BIRDS) and temperature with water-NAA

For temperature and pH_e_ imaging with BIRDS, the anesthetized rats with 9L (n=13), RG2 (n=15), U87 (n=4) and U251 (treated, n=5; not treated, n=6) tumors were prepared with renal ligation to maintain a high TmDOTP^5-^ or TmDOTMA^-^ concentration in the brain**^5,6^**. Since renal ligation stops clearance of the agent, the agent concentration builds up in the microvasculature sufficiently high enough for slow extravasation, which leads to agent accumulation in the extracellular space. In tumors, because of porous immature blood vessels, we expect higher agent accumulation compared to normal tissue. The BIRDS data were acquired using the same circular single-loop ^1^H radio frequency (RF) coil (14 mm diameter) as for the ^1^H-[^13^C] MRS measurements, positioned on top of the animal head. Prior to BIRDS experiments, T_2_-weighted spin-echo MR images were obtained using 11 coronal slices of 128×128 resolution and 1 mm thickness, a field of view (FOV) of 25×25 mm^2^, a TR of 6s and a TE of 40 ms. Following TmDOTP^5-^ or TmDOTMA^-^ infusion, similar MR images were acquired to confirm tumor localization by hypointensity induced by the presence of the paramagnetic agent**^7^**. BIRDS data for both TmDOTP^5-^ and TmDOTMA^-^ experiments were obtained using a 3D 25×25×25 MRSI sequence with a TR of 5ms and a FOV of 25×25×25 mm^3^. The phase encode gradient duration was 160μs, the spectral window was 250kHz and the acquisition time was 4.1ms. Selective excitation of the H2/H3 and H6 protons of TmDOTP^5-^ was achieved using a dual-banded refocused 90° Shinnar-Le Roux (SLR) RF pulse of 35 kHz bandwidth and 90 kHz separation with 205μs duration. For the temperature measurements using TmDOTMA^-^, selective excitation of the CH_3_ peak was achieved using a single band refocused 90° SLR pulse of 40 kHz bandwidth and 205μs duration. The ^1^H spectrum in each voxel was line broadened (500 Hz), phased (zero order), and baseline corrected (first order). The temperature T and pH using TmDOTP^5-^ were calculated in each voxel from the δ_2_, δ_3_ and δ_6_ chemical shifts

$T=a_{0}+\sum_{k=2,3,6} a_{1}^{k}\delta_{k}+\sum_{k=2,3,6} \sum_{j=2,3,6} a_{2}^{kj}\delta_{k}\delta_{j}$ (S2.1a)

${pH}_{e}=b_{0}+\sum_{k=2,3,6} b_{1}^{k}\delta_{k}+\sum_{k=2,3,6} \sum_{j=2,3,6} b_{2}^{kj}\delta_{k}\delta_{j}$ (S2.1b)

where coefficients $a_{0}$_,_ $a_{1}^{k}$, $a_{2}^{kj}$, $b_{0}$_,_ $b_{1}^{k}$ and $b_{2}^{kj}$were obtained from non-linear least-squares fit of temperature or pH as a function of δ_2_, δ_3_ and δ_6_ as previously described**^6^**. The temperature T with TmDOTMA^-^ was calculated from chemical shift δ_CH3_ of the methyl resonance

$T= c_{0}+c_{1}\left( \delta_{CH3}+103 \right)+c_{2}\left( \delta_{CH3}+103 \right)^{2}$ (S2.1c)

where c_0_, c_1_ and c_2_ were 34.45±0.01, 1.460±0.003 and 0.0152±0.0009, respectively, obtained also from non-linear least-squares fit of temperature as function of δ_CH3_**^5^**. The tumor boundary was defined by T_2_-weighted MRI contrast. A voxel was considered inside or outside the tumor if the partial volume occupied by tumor or non-tumor tissue, respectively, was larger than 50%.

In 4 rats with 9L tumors and in 5 rats with U251 tumors, prior to TmDOTP^5-^ infusion for BIRDS, single voxel ^1^H-MRS spectra were acquired separately from tumor and normal tissue using the same LASER technique with VAPOR water suppression**^8^** as for ^1^H-[^13^C] MRS experiments described below. However, in this case the ^1^H-MRS spectra were acquired without ^13^C inversion and with TR = 3s, TE = 25ms and 128 averages. The temperature T was calculated from the difference between the water (δ_water_) and NAA (δ_NAA_) chemical shifts according to the equation**^9^**

$T=286.9-94 \cdot\left( \delta_{water}-\delta_{NAA} \right)$ (S2.1d)

## S2.2 - Cerebral metabolic rate of oxygen consumption (CMR_O2_) with ^1^H-[^13^C] MRS

CMR_O2_ was measured using *in vivo* proton-observed carbon-edited (POCE; ^1^H-[^13^C]) MRS**^10-12^**. The localized ^1^H-[^13^C] MRS data were acquired using a ^1^H/^13^C hybrid radio-frequency (RF) coil comprised of a circular single-loop ^1^H coil (14 mm diameter) positioned on top of the rat head and two orthogonal single loop ^13^C coils (21 mm diameter) driven in quadrature mode and positioned on both sides of the animal head at 45° relative to the ^1^H coil. A T_1_-weighted MRI was acquired and used for tumor delineation and positioning of the ^1^H-[^13^C] MRS voxel inside the tumor or normal tissue. Each rat received about 2 mL of [1,6-^13^C]-D-glucose for a total dose of 8.3 mmol/kg. The [1,6-^13^C]-D-glucose concentration was calculated for each animal based on its weight. During the first 8 minutes the infusion rate was modified manually every 30 seconds to follow a decreasing exponential function and was constant for the remaining of the experiment (~2 hours)**^11^**.

The ^1^H-[^13^C] spectra were obtained from a localized volume of 100µL (5mm×4mm×5mm) from normal tissue (n=4), and from a localized volume in the range of 27-64µL from tumor tissue, depending on the size of the tumor (n=4). The ^1^H-[^13^C] spectra were acquired using localization by adiabatic selective refocusing (LASER) technique with water suppression achieved by variable pulse power and optimized relaxation delays (VAPOR) module**^8^**. Three hundred and eighty four pairs of ^13^C edited/non-edited spectra were acquired during [1,6-^13^C]-D-glucose infusion with a repetition time (TR) of 2.5s and 4 averages, saved separately. The spectra were phased (zero and first order) and frequency aligned using the ^1^H creatine/phosphocreatine peak at 3.0 ppm using Matlab (MathWorks, Inc., Natick, MA). To increase the measurement accuracy, every 24 pairs of aligned spectra was averaged, resulting in a time resolution of ~8 minutes per datapoint. The ^13^C labeled signals were obtained by subtracting the ^13^C edited from the non-edited spectra. The glutamate C4 peak intensity was quantified by LCModel algorithm**^13^**, using a basis set comprised of spectra from 21 metabolites simulated using an echo time (TE) of 22.3ms. The initial slope of the time-dependence of the glutamate C4 peak intensity (**Fig. S1A**) is proportional with the tricarboxylic acid (TCA) cycle flux (V_TCA_)**^11,14^**

CMR_O2_ = 3 × V_TCA_ (S2.2)

where CMR_O2_ was measured in tumors and in normal brain**^14^**.

## S2.3 - Cerebral metabolic rate of glucose consumption (CMR_glc_) with ^19^F-MRSI

The ^19^F MRSI datasets (n=5) were acquired using a single loop circular RF coil (21 mm diameter) positioned on top of the animal head and tunable to both ^1^H and ^19^F frequencies (499.81 and 470.2 MHz, respectively). The FDG infusion rate and total volume infused were calculated for each animal based on its weight to obtain the targeted dose (500 mg/kg). During acquisition, blood samples were collected to measure the FDG concentration in the plasma and to calculate the arterial input function as previously described**^15^**. The ^19^F 8×8 2D MRSI data were obtained using a TR of 0.2s, 128 averages, a 6 mm slice with a field of view of 32×32 mm and a spectral window of 20 kHz. The ^19^F signals were measured in a voxel covering the tumor and in one covering normal tissue (from the contralateral hemisphere). The ^19^F spectra were line broadened (10 Hz), baseline (first order) and phase (zero order) corrected. Nominal and effective voxel volumes were 96μL and 112μL, respectively. Reversible FDG transport into the brain tissue and its phosphorylation were described using a model consisting of three pools, FDG in blood plasma (FDG_p_), FDG in brain tissue (FDG_t_) and 2-fluoro-2-deoxy-D-glucose-6-phosphate (FDG-6P), present only in brain tissue**^15^**. The model uses the ratio R_m_ of FDG-6P and FDG signals and the FDG concentration in the blood plasma to determine the FDG_t_ and FDG-6P concentrations in the brain tissue (**Fig. S1B**), and to calculate the conversion rate of FDG_t_ to FDG-6P (CMR_glc_) according to**^15^**

CMR_glc_ = 0.094 × R_m_ × [FDG_t_] min^-1^ (S2.3)

## S2.4 - Cerebral blood flow (CBF) with pulsed arterial spin labeling (PASL)

The longitudinal relaxation time T_1_ mapping and CBF data (n=7) was obtained using a hybrid ^1^H volume RF coil (80 mm diameter) for transmission combined with a ^1^H surface RF coil (35 mm diameter) for acquisition. T_1_ maps were obtained before each CBF measurement using a spin-echo sequence with an echo time (TE) of 9ms and 5 different TR values of 0.4, 0.7 ,1 ,2 and 6 s. Eleven coronal slices of 1mm thickness were acquired at 128 × 128 resolution and field of view of 25 mm × 25 mm. The T_1_ values were calculated by fitting the MR intensities versus TR to a single exponential function. The CBF was measured using PASL with echo-planar imaging (EPI). Alternative images with global/slice selective inversion were obtained using an adiabatic inversion pulse. The spin-echo EPI images were obtained with a TR of 36s, TE of 36ms, 3 coronal slices of 1mm each. The image resolution was 64 × 64 with a field of view of 25 mm × 25 mm. CBF was calculated from the difference between the image intensity obtained with global inversion (*S_ginv_*) and the one with slice selective inversion (*S_sinv_*) according to**^16^**:

$CBF=\frac{\lambda}{T_{1app}}\frac{S_{ginv}-S_{sinv}}{2S_{ginv}}$ (S2.4)

where λ = 0.95 is the blood-brain partition coefficient for water and T_1app_ is the apparent longitudinal relaxation time of brain tissue (measured separately). The T_1_ maps were used to separate the tumors from normal tissue. Because the water T_1_ was different inside the tumors compared to normal tissue, two PASL experiments were obtained with different inversion-recovery times (TIRs): 2.1s for normal tissue and 2.6s for tumors. A joined CBF map was generated by combining the CBF images measured using these two different TIR values (**Fig. S2**).

## S2.5 - Measurement of cerebrovascular reactivity (CVR) with hypercapnia

CVR data (n=4) were obtained using the same hybrid ^1^H volume/surface RF coil as for the CBF measurements. The cerebrovascular reactivity was assessed by increasing the inhaled CO_2_ from 0% to 5% (hypercapnia). 720 EPI images were acquired using a gradient-echo sequence with a TR of 1s, TE of 16ms, 3 slices of 1mm each. The image resolution was 64 × 64 with a field of view of 25 mm × 25 mm. The hypercapnia protocol involved 3 minutes baseline (180 images) followed by 3 minutes exposure to 5% CO_2_ (180 images). After the CO_2_ exposure, 360 EPI images were acquired for 6 minutes to monitor the signal readjustment towards the baseline value. Two CVR datasets were acquired and averaged for each animal. The relative response during hypercapnia (i.e. the fractional change in the MR signal relative to the pre-stimulus state) was estimated by normalizing the absolute MR time course to the average MR signal for the pre-stimulus state (first 180 images in the series). The time course of the average normalized MR intensity during hypercapnia challenge was measured inside the tumor and in the normal brain tissue, where the tumor region was identified by the hypointensity in the T_2_-weighted MR image.

## S2.6 - Measurement of tissue cellularity apparent diffusion coefficient (ADC) imaging

The ADC map was obtained using the same hybrid ^1^H volume/surface RF coil as for the CBF measurements. The ADC experiments were performed using a spin-echo diffusion-weighted sequence with 5 different b-values: 500, 700, 1000 2000 and 3000 s/mm^2^, 5 images with no diffusion gradients, a TR of 3s, a TE of 26ms, 3 slices of 1mm thickness, an FOV of 25mm × 25mm and 64 × 64 resolution. The ADC value in each pixel was calculated by non-linear least-squares fit of image intensity versus b-value according to a single exponential decay function.

**Table S1.** Definitions, values, and units for the parameters used for temperature simulations using the steady-state bioheat **eq. 2** in main text. CBF, CMR_O2_, CMR_glc_, T_art_ and T_env_ were measured in this study. All the other parameters were taken from literature**^17-20^**. See **Table S2** for ratios of CMR_O2_, CMR_glc_, and CBF in tumor and normal tissue.

|  | **Definition** | **Value** | **Unit** |
| --- | --- | --- | --- |
| T_ts_(x,y) | tissue temperature at Cartesian coordinates x,y | - | °C |
| T_art_ | arterial blood temperature | 37 | °C |
| T_env_ | environmental temperature | 22 | °C |
| k | thermal conductivity of brain tissue | 0.56 | J/s/m/°C |
| h_n_ | heat transfer from normal brain tissue through skull to environment | 8 | J/s/m^2^/g/°C |
| h_t_ | heat transfer from tumor tissue through skull to environment | 20 | J/s/m^2^/g/°C |
| ΔH_0_ | enthalpy of oxidative phosphorylation (including glycolysis) | 0.47 | J/μmol |
| ΔH_b_ | energy required for O_2_ release from hemoglobin | 0.028 | J/μmol |
| ΔH_glyc_ | enthalpy of glycolysis | 0.061 | J/μmol |
| ρ_ts_ | tissue density | 1.04 | g/mL |
| ρ_bl_ | blood density | 1.06 | g/mL |
| c_bl_ | specific heat coefficient of blood | 3.9 | J/g/°C |
| A | surface area of a voxel element | 10^-6^ | m^2^ |
| CMR_O2,n_ | cerebral metabolic rate of oxygen consumption in normal brain tissue | 1.50 | μmol/g/min |
| CMR_O2,t_ | cerebral metabolic rate of oxygen consumption in the tumor | 0.31 | μmol/g/min |
| CMR_glc,n_ | cerebral metabolic rate of glucose consumption in normal brain tissue | 0.25 | μmol/g/min |
| CMR_glc,t_ | cerebral metabolic rate of glucose consumption in the tumor | 1.06 | μmol/g/min |
| CBF_n_ | cerebral blood flow in normal brain tissue | 0.50 | mL/g/min |
| CBF_t_ | cerebral blood flow in the tumor | 0.32 | mL/g/min |

**Table S2.** Ratios between parameters measured inside the tumor and normal tissue (see **Figs. 1** and **2)**. See **Table S1** for values of CMR_O2_, CMR_glc_, CBF, and h in tumor and normal tissue. See **Fig. S4** for ADC differences in tumor normal tissue, where 30% change in ADC corresponds to 250% change in cellular density which is related to h**^21^**.

| **Parameter** | **Ratio (tumor / normal tissue)** |
| --- | --- |
| CMR_O2_ ratio | 0.21 (= CMR_O2,t_ / CMR_O2,n_ ) |
| CMR_glc_ ratio | 4.24 (= CMR_glc,t_ / CMR_glc,n_ ) |
| CBF ratio | 0.64 (= CBF_t_ / CBF_n_ ) |
| H ratio | 2.5 (=h_t_ / h_n_ ) |

**Table S3.** Parameters in **Fig. 3** for temperature simulations, calculated average temperatures for normal brain (T_n_) and tumor (T_t_), and temperature gradient between normal and tumor tissue ΔT. Definitions, values, and units for parameters used in the simulations (**eq. 2** in main text) are given in **Table S1**. For all simulations, the ratios between the tumor and normal tissue parameters were kept constant and equal to those measured in this study (**Table S2**). The thermal conductivity k and the extracranial environmental temperature T_env_ were considered the same inside/outside the tumor. Parameters measured in this study are indicated by *.

| **CBF_n_** | **CBF_t_** | **CMR_O2,n_** | **CMR_O2,t_** | **CMR_glc,n_** | **CMR_glc,t_** | **h_n_** | **h_t_** | **k** | **T_env_** | **T_n_** | **T_t_** | **ΔT** |
| --- | --- | --- | --- | --- | --- | --- | --- | --- | --- | --- | --- | --- |
| 0.25 | 0.16 | 1.5 | 0.3 | 0.25 | 1.06 | 8 | 20 | 0.564 | 22 | 32.47±0.21 | 31.71±0.12 | 0.77 |
| 0.5* | 0.32 | 1.5 | 0.3 | 0.25 | 1.06 | 8 | 20 | 0.564 | 22 | 34.25±0.20 | 33.45±0.14 | 0.80 |
| 1 | 0.64 | 1.5 | 0.3 | 0.25 | 1.06 | 8 | 20 | 0.564 | 22 | 35.46±0.17 | 34.71±0.14 | 0.75 |
| 2 | 1.28 | 1.5 | 0.3 | 0.25 | 1.06 | 8 | 20 | 0.564 | 22 | 36.18±0.12 | 35.56±0.12 | 0.62 |
| 0.5 | 0.32 | 0.75 | 0.15 | 0.25 | 1.06 | 8 | 20 | 0.564 | 22 | 34.13±0.20 | 33.35±0.13 | 0.78 |
| 0.5 | 0.32 | 1.5* | 0.3 | 0.25 | 1.06 | 8 | 20 | 0.564 | 22 | 34.25±0.20 | 33.45±0.14 | 0.80 |
| 0.5 | 0.32 | 3 | 0.6 | 0.25 | 1.06 | 8 | 20 | 0.564 | 22 | 34.50±0.21 | 33.66±0.14 | 0.84 |
| 0.5 | 0.32 | 6 | 1.2 | 0.25 | 1.06 | 8 | 20 | 0.564 | 22 | 34.99±0.23 | 34.09±0.15 | 0.91 |
| 0.5 | 0.32 | 1.5 | 0.3 | 0.125 | 0.525 | 8 | 20 | 0.564 | 22 | 34.25±0.20 | 33.45±0.14 | 0.80 |
| 0.5 | 0.32 | 1.5 | 0.3 | 0.25* | 1.06 | 8 | 20 | 0.564 | 22 | 34.25±0.20 | 33.45±0.14 | 0.80 |
| 0.5 | 0.32 | 1.5 | 0.3 | 0.5 | 2.12 | 8 | 20 | 0.564 | 22 | 34.26±0.20 | 33.46±0.13 | 0.80 |
| 0.5 | 0.32 | 1.5 | 0.3 | 1 | 4.24 | 8 | 20 | 0.564 | 22 | 34.28±0.20 | 33.48±0.13 | 0.79 |
| 0.5 | 0.32 | 1.5 | 0.3 | 0.25 | 1.06 | 2 | 5 | 0.564 | 22 | 36.41±0.07 | 36.15±0.04 | 0.27 |
| 0.5 | 0.32 | 1.5 | 0.3 | 0.25 | 1.06 | 4 | 10 | 0.564 | 22 | 35.61±0.12 | 35.14±0.08 | 0.48 |
| 0.5 | 0.32 | 1.5 | 0.3 | 0.25 | 1.06 | 8* | 20 | 0.564 | 22 | 34.25±0.20 | 33.45±0.14 | 0.80 |
| 0.5 | 0.32 | 1.5 | 0.3 | 0.25 | 1.06 | 16 | 40 | 0.564 | 22 | 32.22±0.30 | 31.02±0.20 | 1.20 |
| 0.5 | 0.32 | 1.5 | 0.3 | 0.25 | 1.06 | 8 | 20 | 0.282 | 22 | 34.24±0.28 | 32.99±0.23 | 1.25 |
| 0.5 | 0.32 | 1.5 | 0.3 | 0.25 | 1.06 | 8 | 20 | 0.564* | 22 | 34.25±0.20 | 33.45±0.14 | 0.80 |
| 0.5 | 0.32 | 1.5 | 0.3 | 0.25 | 1.06 | 8 | 20 | 1.128 | 22 | 34.29±0.14 | 33.81±0.08 | 0.48 |
| 0.5 | 0.32 | 1.5 | 0.3 | 0.25 | 1.06 | 8 | 20 | 2.256 | 22 | 34.34±0.08 | 34.07±0.04 | 0.27 |
| 0.5 | 0.32 | 1.5 | 0.3 | 0.25 | 1.06 | 8 | 20 | 0.564 | 19 | 33.65±0.24 | 32.70±0.16 | 0.96 |
| 0.5 | 0.32 | 1.5 | 0.3 | 0.25 | 1.06 | 8 | 20 | 0.564 | 22* | 34.25±0.20 | 33.45±0.14 | 0.80 |
| 0.5 | 0.32 | 1.5 | 0.3 | 0.25 | 1.06 | 8 | 20 | 0.564 | 25 | 34.85±0.16 | 34.21±0.11 | 0.65 |
| 0.5 | 0.32 | 1.5 | 0.3 | 0.25 | 1.06 | 8 | 20 | 0.564 | 28 | 35.45±0.13 | 34.96±0.08 | 0.49 |

**Table S4.** The tumor/normal parameters ratios used in **Fig. S3** for temperature simulations, the calculated average temperatures for normal brain (T_n_) and tumor (T_t_), and the temperature gradient between normal and tumor tissue (ΔT). The definitions, values and units for the parameters used in the simulations (**eq. 2** in main text) are given in **Table S1**. See **Table S2** for values of CMR_O2_, CMR_glc_, CBF, and h in tumor and normal tissue, which gives the respective ratios. For all simulations shown in this table, the normal brain parameters were kept constant and equal to those measured in this study. The thermal conductivity k and the environmental temperature T_env_ were considered the same inside and outside the tumor and therefore not studied. The tumor/normal ratios measured experimentally in this study are indicated by *. Negative ΔT values indicate that the tumor temperature is larger than normal tissue temperature.

| CBF ratio | CMR_O2_ ratio | CMR_glc_ ratio | h ratio | T_n_ (°C) | T_t_ (°C) | ΔT (°C) |
| --- | --- | --- | --- | --- | --- | --- |
| 0.06 | 0.2 | 4.2 | 2.5 | 34.15±0.28 | 33.03±0.19 | 1.12 |
| 0.6* | 0.2 | 4.2 | 2.5 | 34.25±0.20 | 33.45±0.14 | 0.80 |
| 6 | 0.2 | 4.2 | 2.5 | 34.70±0.15 | 35.27±0.09 | -0.57 |
| 0.6 | 0.02 | 4.2 | 2.5 | 34.25±0.21 | 33.44±0.14 | 0.81 |
| 0.6 | 0.2* | 4.2 | 2.5 | 34.25±0.20 | 33.45±0.14 | 0.80 |
| 0.6 | 2 | 4.2 | 2.5 | 34.28±0.18 | 33.56±0.12 | 0.72 |
| 0.6 | 0.2 | 0.42 | 2.5 | 34.25±0.21 | 33.45±0.14 | 0.81 |
| 0.6 | 0.2 | 4.2* | 2.5 | 34.25±0.20 | 33.45±0.14 | 0.80 |
| 0.6 | 0.2 | 42 | 2.5 | 34.27±0.19 | 33.51±0.13 | 0.76 |
| 0.6 | 0.2 | 4.2 | 0.25 | 34.56±0.04 | 34.71±0.02 | -0.15 |
| 0.6 | 0.2 | 4.2 | 2.5* | 34.25±0.20 | 33.45±0.14 | 0.80 |
| 0.6 | 0.2 | 4.2 | 25 | 32.84±1.31 | 27.81±0.76 | 5.03 |


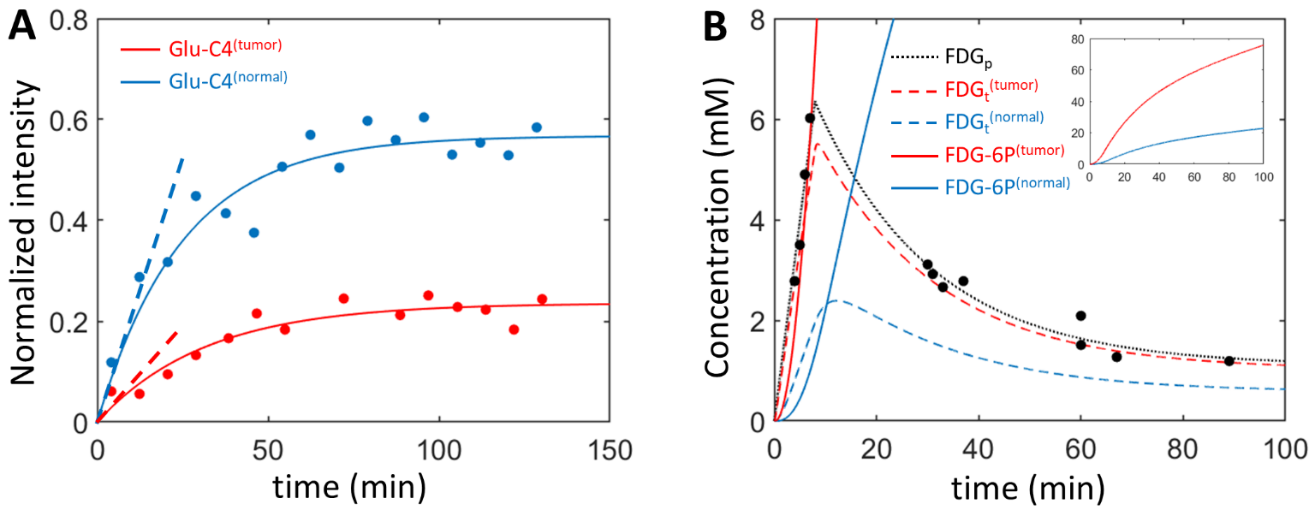


**Figure S1.** **Time courses of ^1^H-[^13^C] MRS (or POCE) and ^19^F-MRSI data to determine CMR_O2_ and CMR_glc_ in 9L tumors and normal tissue. (A)** CMR_O2_ was calculated using the time dependence of the glutamate C4 peak (**Methods S2.2**) measured inside tumor (red) and in normal tissue (blue). The solid line represents the fit of glutamate C4 signal to a one phase association function described by a single exponential. The initial slope, indicated in each case by a dashed line, is proportional with the TCA cycle flux V_TCA_, allowing comparison of CMR_O2_ values inside the tumor with those in normal tissue. **(B)** The time dependence of the ^19^F FDG signal in the plasma (FDG_p_, dotted black line) and the ratio between ^19^F signals of FDG-6P and FDG can be used to calculate the FDG and FDG-6P concentrations in the brain tissue measured inside tumors (continuous and dashed red lines, respectively) and in normal tissue (continuous and dashed blue lines, respectively). The black circles represent the FDG concentration in the plasma extracts measured at various time points during the FDG infusion. The inset in **(B)** represents the accumulation of FDG-6P in the brain tissue inside the tumor (red line) and in normal tissue (blue line) indicating a faster conversion of FDG to FDG-6P in the 9L tumors compared to normal tissue. CMR_glc_ was calculated using these ^19^F signals (**Methods S2.3**).


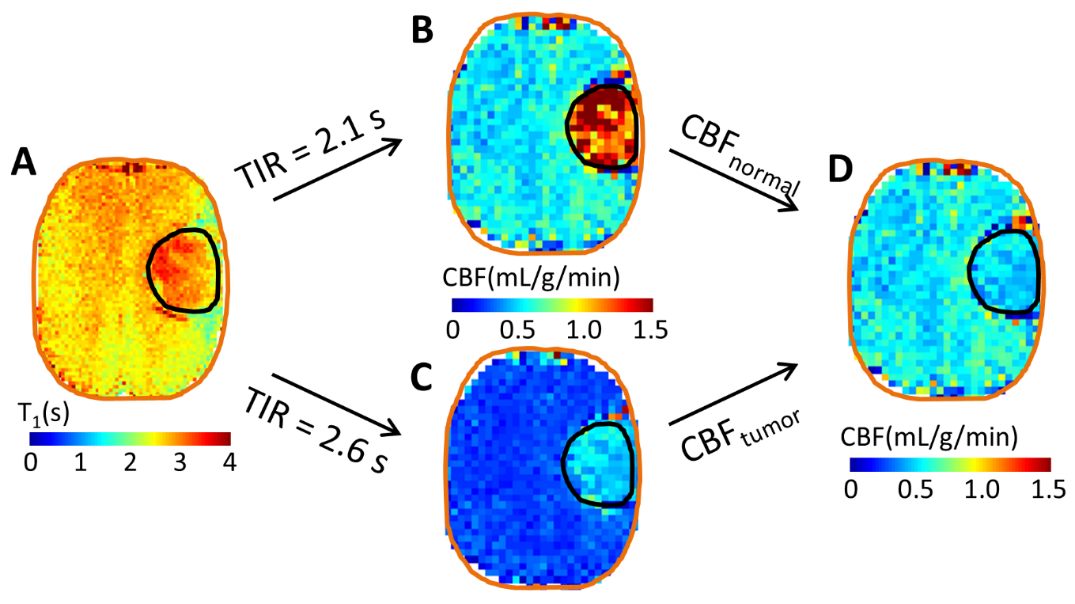


**Figure S2.** **Reconstruction of CBF maps in rat brain using two inversion recovery times (TIR) from PASL experiments.** **(A)** T_1_ map of rat brain with the tumor region identified by hyperintensity of T1 values (tumor = black outline, brain = orange outline). The T_1_ map was used to separate the tumor region from the normal tissue. PASL was used to obtain two CBF maps using **(B)** TIR = 2.1s and **(C)** TIR = 2.6s because of the different T_1_ values for normal and tumor tissues. **(D)** Because T_1_ values are different in tumor versus normal tissues, affecting the CBF estimation, a joint CBF map was generated using the values obtained with TIR = 2.1s for normal tissue and with TIR = 2.6s for tumor tissue (see **Methods S2.4**).

**
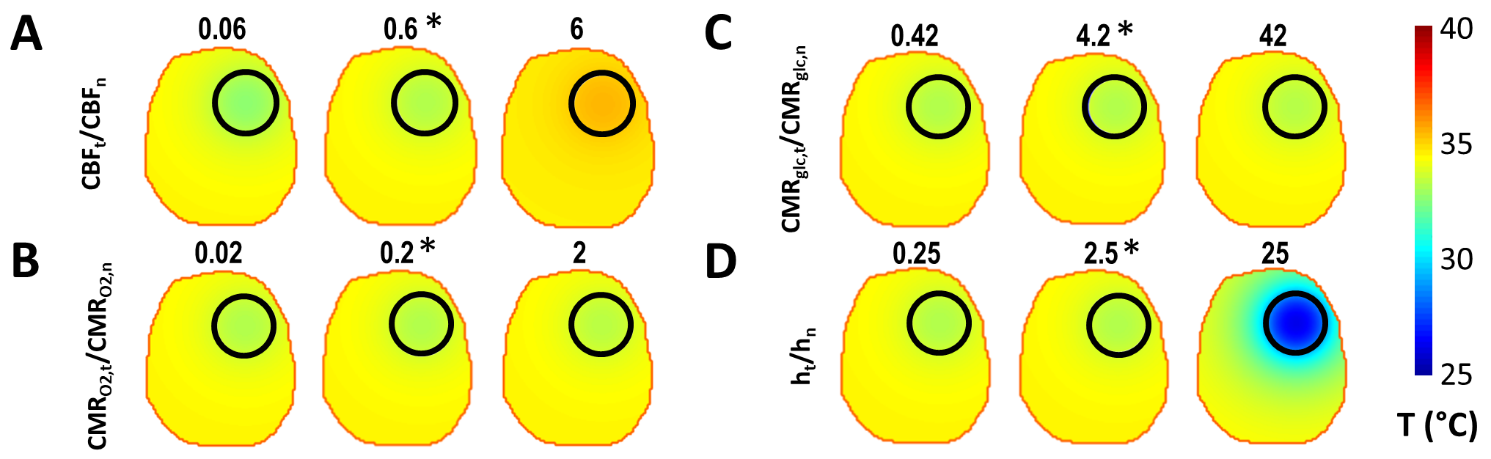
**

**Figure S3. Simulations of brain T maps using a 2D steady-state heat transfer model where the tumor/normal ratio for each parameter was modified from the measured value.** The T maps were calculated using the Pennes bioheat equation, where physiological parameters (CBF, CMR_O2_, and CMR_glc_), biophysical constants (h and k), and the environmental temperature (T_env_) influenced the brain T (see **Section 2.3** and **eq. 2** in main text). The T maps represent a horizontal section of a rat brain, which includes a spherical tumor (5 mm diameter) in the right frontoparietal region (tumor = black outline, brain = orange outline). To study the effect of individually changing the tumor/normal ratio for each parameter while keeping all normal tissue and the rest of tumor parameter values constant and equal to the measured values, we calculated T_n_, T_t_ and ΔT for the following conditions: **(A)** CBF_t_/CBF_n_ from 0.06 to 6, **(B)** CMR_O2,t_/CMR_O2,n_ from 0.02 to 2, **(C)** CMR_glc,t_/CMR_glc,n_ from 0.42 to 42 and **(D)** h_t_/h_n_ from 0.25 to 25. The k and T_env_ values were considered the same inside and outside the tumor, and the effect of their variation is shown in **Fig. 3E-F**. The corresponding tumor/normal ratio is indicated at the top of each map. The T maps that correspond to the tumor/normal ratios measured experimentally in this study are indicated by *. All T_n_, T_t_ and ΔT values obtained from these simulations are given in **Table S3**.

**
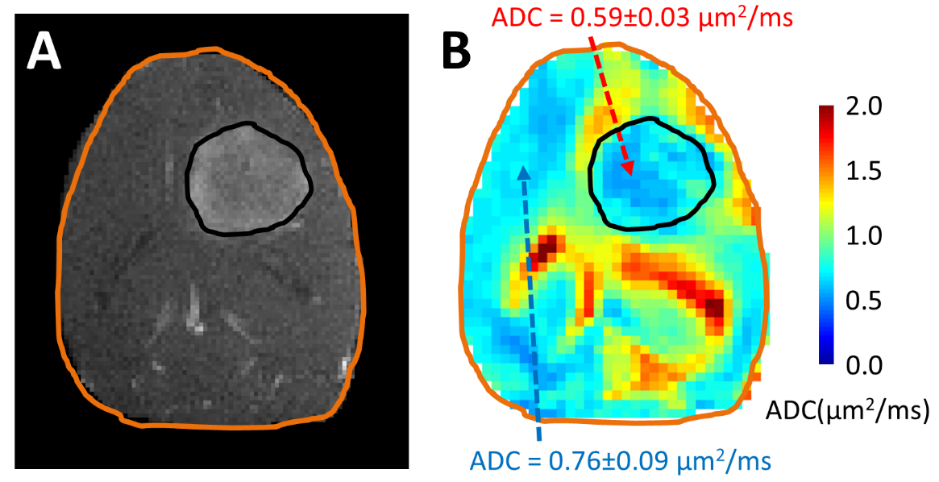
**

**Figure S4. Example of ADC measurement in a rat brain with a U251 tumor. (A)** T_1_-weighted MR image of rat brain, with the tumor region identified by hyperintensity (tumor = black outline, brain = orange outline). **(B)** The ADC map was calculated form a series of diffusion weighted images with various b-values (0 to 3000 s/mm²). The lower ADC measured inside the tumor (0.59±0.03 µm^2^/ms) compared to normal tissue (0.76±0.09 µm^2^/ms) indicates a more restricted water diffusion due to higher cellular density of tumors, where 30% change in ADC corresponds to 250% change in cellular density^21^.

**
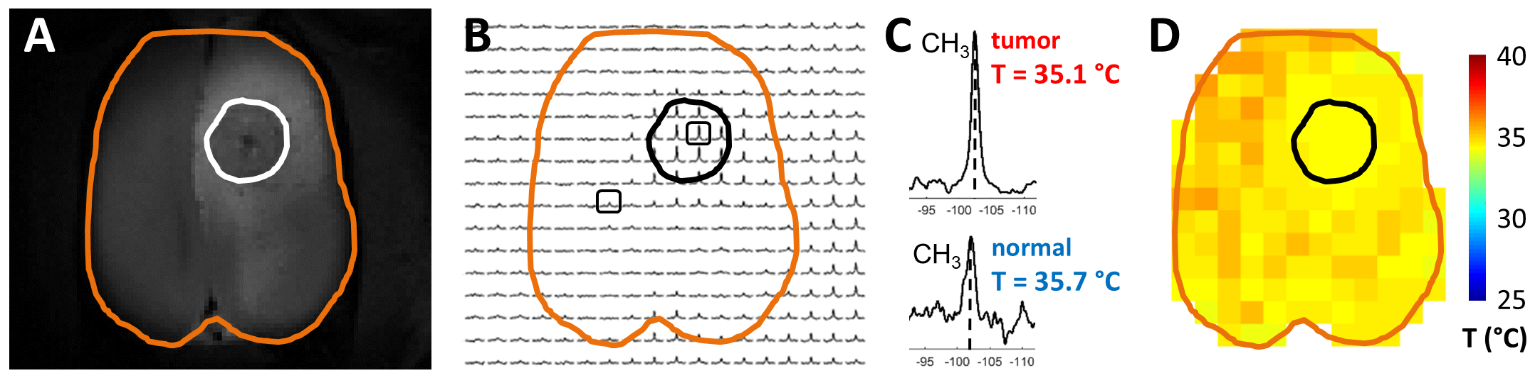
**

**Figure S5.** **Example of** **brain temperature imaging by BIRDS with TmDOTMA^-^ in rat brain with a U251 tumor. (A)** MRI of rat brain after TmDOTMA^-^ infusion, where the tumor region was identified with hypointensity in a T_2_-weighted image (tumor = black outline, brain = orange outline). **(B)** 3D MRSI data for the slice shown in **A** reveals varying TmDOTMA^-^ levels throughout the brain. **(C)** Examples of ^1^H spectra from tumor (top) and normal tissue (bottom), showing the CH_3_ peak of TmDOTMA^-^. The absolute temperature T was calculated from the chemical shift of the CH_3_ peak (see **Methods S2.1**; **eq. 2.1c**) and shows a lower temperature inside the tumor (T = 35.1 °C) compared to normal tissue (T = 35.7 °C). **(D)** T map using BIRDS with TmDOTMA^-^ for the same slice shown in **A** and **B**.

**
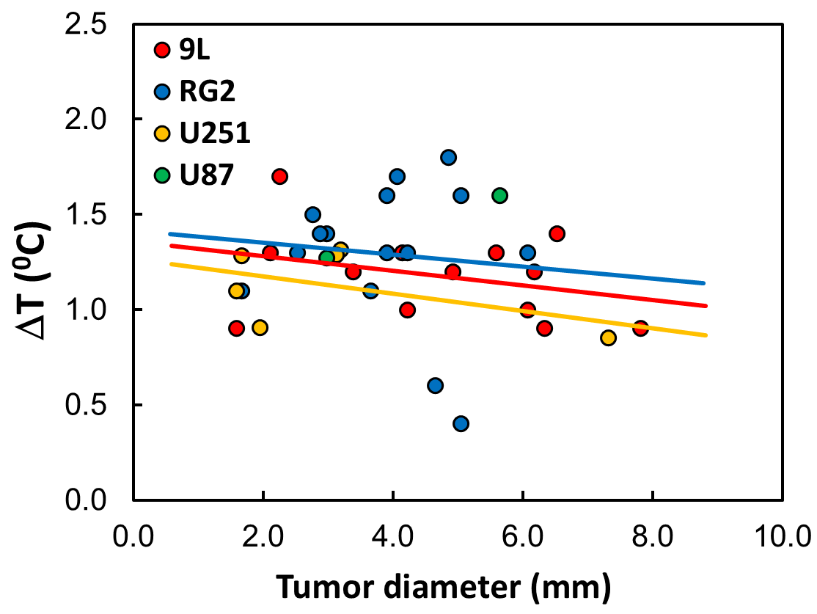
**

**Figure S6. Correlation between tumor size and** **temperature gradient ΔT between normal and tumor tissue for various tumor types.** A weak to moderate correlation (0.1<r<0.5) was observed between ΔT and tumor diameter for 9L (red, r=0.32, n=13), RG2 (blue, r=0.10, n=15), U251 (yellow, r=0.48, n=6) and U87(green, r=not available, n=2). The tumor diameter was estimated from the tumor area, measured using the T_2_-weighted MR images, and assuming a circular tumor shape. For U87 tumors, the r-value was not calculated because only 2 values were available, leading to a perfect (and false) correlation (r=1).

**REFERENCES**

**1.** Kimler BF. The 9L rat brain tumor model for pre-clinical investigation of radiation-chemotherapy interactions. *J Neurooncol.* 1994; 20(2):103-109.

**2.** Wechsler W, Kleihues P, Matsumoto S, et al. Pathology of experimental neurogenic tumors chemically induced during prenatal and postnatal life. *Annals of the New York Academy of Sciences.* 1969; 159(2):360-408.

**3.** Pontén J, Macintyre EH. Long term culture of normal and neoplastic human glia. *Acta Pathol Microbiol Scand.* 1968; 74(4):465-486.

**4.** Bigner DD, Bigner SH, Pontén J, et al. Heterogeneity of Genotypic and phenotypic characteristics of fifteen permanent cell lines derived from human gliomas. *J Neuropathol Exp Neurol.* 1981; 40(3):201-229.

**5.** Coman D, Trubel HK, Hyder F. Brain temperature by Biosensor Imaging of Redundant Deviation in Shifts (BIRDS): comparison between TmDOTP5- and TmDOTMA. *NMR in biomedicine.* 2010; 23(3):277-285.

**6.** Coman D, Trubel HK, Rycyna RE, Hyder F. Brain temperature and pH measured by (1)H chemical shift imaging of a thulium agent. *NMR in biomedicine.* 2009; 22(2):229-239.

**7.** Coman D, Huang Y, Rao JU, et al. Imaging the intratumoral-peritumoral extracellular pH gradient of gliomas. *NMR in biomedicine.* 2016; 29(3):309-319.

**8.** de Graaf RA. *In vivo NMR Spectroscopy - Principles and Techniques (2nd Edition)*. Chichester, West Sussex, England: Wiley, John & Sons, Incorporated; 2007.

**9.** Cady EB, D'Souza PC, Penrice J, Lorek A. The estimation of local brain temperature by in vivo 1H magnetic resonance spectroscopy. *Magn Reson Med.* 1995; 33(6):862-867.

**10.** Rothman DL, Behar KL, Hetherington HP, et al. 1H-Observe/13C-decouple spectroscopic measurements of lactate and glutamate in the rat brain in vivo. *Proceedings of the National Academy of Sciences of the United States of America.* 1985; 82(6):1633-1637.

**11.** Fitzpatrick SM, Hetherington HP, Behar KL, Shulman RG. The flux from glucose to glutamate in the rat brain in vivo as determined by 1H-observed, 13C-edited NMR spectroscopy. *J Cereb Blood Flow Metab.* 1990; 10:170-179.

**12.** Rothman D, Novotny E, Shulman G, et al. 1H-[13C] NMR measurements of [4-13C]glutamate turnover in human brain. *Proc Natl Acad Sci USA.* 1992; 89:9603-9606.

**13.** Provencher SW. Estimation of metabolite concentrations from localized in vivo proton NMR spectra. *Magn Reson Med.* 1993; 30(6):672-679.

**14.** Hyder F, Chase JR, Behar KL, et al. Increased tricarboxylic acid cycle flux in rat brain during forepaw stimulation detected with ^1^H[^13^C]NMR. *Proceedings of the National Academy of Sciences of the United States of America.* 1996; 93(15):7612-7617.

**15.** Coman D, Sanganahalli BG, Cheng D, McCarthy T, Rothman DL, Hyder F. Mapping phosphorylation rate of fluoro-deoxy-glucose in rat brain by (19)F chemical shift imaging. *Magnetic resonance imaging.* 2014; 32(4):305-313.

**16.** Calamante F, Thomas DL, Pell GS, Wiersma J, Turner R. Measuring cerebral blood flow using magnetic resonance imaging techniques. *J Cereb Blood Flow Metab.* 1999; 19(7):701-735.

**17.** Zhu M, Ackerman JJ, Sukstanskii AL, Yablonskiy DA. How the body controls brain temperature: the temperature shielding effect of cerebral blood flow. *J Appl Physiol.* 2006; 101(5):1481-1488.

**18.** Trubel HK, Sacolick LI, Hyder F. Regional temperature changes in the brain during somatosensory stimulation. *J Cereb Blood Flow Metab.* 2006; 26(1):68-78.

**19.** Yablonskiy DA, Ackerman JJ, Raichle ME. Coupling between changes in human brain temperature and oxidative metabolism during prolonged visual stimulation. *Proceedings of the National Academy of Sciences of the United States of America.* 2000; 97(13):7603-7608.

**20.** Van Leeuwen GM, Hand JW, Lagendijk JJ, Azzopardi DV, Edwards AD. Numerical modeling of temperature distributions within the neonatal head. *Pediatr Res.* 2000; 48(3):351-356.

**21.** Chang PD, Malone HR, Bowden SG, et al. A Multiparametric Model for Mapping Cellularity in Glioblastoma Using Radiographically Localized Biopsies. *American Journal of Neuroradiology.* 2017; 38(5):890-898.
